# Pollinator abundance shapes sexual selection in an angiosperm

**DOI:** 10.1371/journal.pbio.3003579

**Published:** 2026-10-27

**Authors:** Estelle Barbot, Mathilde Dufaÿ, Tim Janicke, Benoit Lapeyre, Denis Orcel, François Rousset, Jeanne Tonnabel

**Affiliations:** 1 ISEM, University of Montpellier, CNRS, IRD, Montpellier, France; 2 CEFE, University of Montpellier, CNRS, EPHE, IRD, Montpellier, France; The Australian National University, AUSTRALIA

## Abstract

Sexual selection is a central force in evolutionary biology that has been shown to shape phenotypes and population dynamics in animals. While this selective force is increasingly recognized in flowering plants, theory predicts that anisogamy generates sex differences in the fitness benefits of acquiring additional mates—a pattern well supported in animals but with limited tests in plants. Plant studies rely solely on genetic parentage analyses to estimate mate numbers, thereby conflating performance in accessing mates with success in gamete fertilization. Pollinators add an ecological layer, as their behavior and abundance can influence competition for access to mates and their gametes. Here, we disentangle consecutive episodes of sexual selection in the hermaphroditic plant *Brassica rapa* by combining direct observations of *Bombus terrestris* movements with genetic paternity analyses in experimental populations, thus providing independent estimates of the observational number of mates acquired and realized fertilization success. Our approach identifies pollen competition for ovules as the primary arena for sexual selection in *B. rapa*. Competition for access to mates constitutes a secondary force and was found to be stronger in the male compared to the female sex function, as predicted by classic theory. Experimentally reduced pollinator abundance modulated the balance between pre- and post-pollination sexual selection, increasing the opportunity for selection on mate acquisition. Ignoring pollinator movements led to biased estimates of pre-pollination sexual selection. Our findings demonstrate that sexual selection in plants unfolds across multiple, sequential episodes shaped by ecological context.

## Abstract 2

What impact does pollinator abundance and behavior have on sexual selection in flowering plants? This study uses bumblebees and Brassica rapa to show that pollen competition for ovules is the primary arena for sexual selection.

## Introduction

Sexual selection is a cornerstone of evolutionary biology, providing a framework to explain the diversity of reproductive strategies observed across the animal kingdom [1,2]. While manifold studies have supported Darwin’s foundational ideas centered on the role of competition for access to sexual partners [3–5], modern developments in the field have established that sexual selection extends beyond mate acquisition in the pre-copulatory phase and encompasses competition for fertilizing gametes in the post-copulatory phase [6–9]. Research on sexual selection in flowering plants has similarly focused on processes occurring during pollen dispersal to mates (pre-pollination) and during pollen competition for ovule fertilization (post-pollination). During the pre-pollination phase, selection over the ability to attract pollinators, the positioning of pollen on the bodies of pollinators, or the export of pollen via wind is expected to shape diverse plant phenotypes, in ways that are functionally analogous to certain components of sexual selection in animals, such as selection on copulation rate [10–12]. In the post-pollination phase, competition among pollen donors influences the evolution of pollen traits, paralleling sperm competition in animals [6,8,13]. By contrast, selection for mate acquisition during the pre-pollination phase—a central component of sexual selection research—has so far received comparatively little attention in plants [11,12,14].

Conceptual thinking about sexual selection has developed around the core idea that selection for accessing mates applies to most sexually reproducing organisms [15,16]. Central to this framework is a prediction derived from anisogamy: because one sex produces smaller, more numerous gametes (by definition the male sex), whereas the other produces larger, fewer gametes (the female sex), the fitness returns from acquiring additional mates should be greater for males [15,17]. These sex differences in the benefit of mate acquisition have been found to prevail across the animal tree of life [3], even though sexual selection in females is potentially more widespread than initially envisioned by the pioneers in the field [18]. Attempts to quantify sexual selection for access to mates in flowering plants remain scarce, but existing studies have converged with findings in animals, showing that males obtain stronger fitness benefits from increased access to sexual partners [19–22]. Yet, these first quantitative approaches estimating the strength of sexual selection for accessing mates in angiosperms failed to incorporate the behavior of a plant-specific third-party vector—the pollinator, which mediates pollen dispersal in 87.5% of angiosperm species [23]. Integrating pollinator movements between plants into studies on sexual selection is critical to fill this knowledge gap and to disentangle the distinct episodes of selection that occur throughout plant reproduction—from gamete transfer to mates during the pre-pollination phase to ovule fertilization in the post-pollination phase.

In insect-pollinated plants, transport of gametes between sessile sexual partners depends on pollinators such that ecological factors modulating pollinator behavior and abundance are prone to influence the operation of sexual selection. While pollinator abundance has been documented to play a prominent role in plant reproductive biology in terms of seed production or selfing rates [24–28], we have virtually no knowledge of its effect on sex-specific patterns of sexual selection for access to mates. This gap contrasts with the abundant animal literature demonstrating that ecological factors, such as population density, can affect the strength and direction of sexual selection. We hypothesized that variation in pollinator abundance is likely to alter the relative importance of pre- and post-pollination episodes of sexual selection by affecting both the baseline probability of accessing a mate and the level of competition among pollen donors once mates have been accessed. At low pollinator abundance, both sexual functions become more limited by the reduced transport of pollen between mates, which is expected to heighten the importance of the pre-pollination episode of sexual selection to access more mates [29]. Conversely, a high pollinator abundance may intensify post-pollination mechanisms by increasing the quantity and diversity of pollen deposited on stigmas, leading to greater competition among deposited pollen from different donors for fertilizing ovules [30]. The combination of higher pollen loads and stronger polyandry could lead to intensified filtering of more competitive pollen donors through male-male competition and/or cryptic female choice. Previous attempts at quantifying sexual selection in plants have relied exclusively on paternity analyses to estimate the number of sexual partners [19–22], which precludes the ability to disentangle the contribution of accessing mates versus ovules and therefore confounds the outcome of pre- and post-pollination episodes of sexual selection [31]. To explore the hypothesized role of pollinator abundance on the relative importance of pre- and post-pollination sexual selection, it is essential to estimate the number of mates accessed through pollinator movements independently of the ability of pollen donors to fertilize ovules.

We quantified sexual selection at key steps of reproduction in an insect-pollinated plant and tested experimentally how pollinator abundance affects the operation of sexual selection at both pre- and post-pollination episodes in each sex function of the hermaphroditic plant *Brassica rapa* pollinated by its main pollinator *Bombus terrestris* [25,32,33]. To decompose sexual selection episodes, we performed exhaustive monitoring of pollinator movements at the flower scale in 16 experimental populations exposed to three levels of pollinator abundance, and genotyped the resulting seeds for paternity assignment. The monitoring of pollinator movements enabled the estimation of a key variable in the quantification of sexual selection—the observational number of mates—defined as the number of sexual partners of a focal plant, accessed in the pre-pollination phase by pollinators exporting gametes from the focal plant in the male role, or importing gametes to the focal plant in the female role. Our measure of the observational number of mates thus provides an estimate for potential mates with whom an individual may reproduce, in contrast to the genetic number of mates corresponding to realized sexual partners with which offspring were successfully produced. As the inferred number of mates depends on assumptions about pollen carry-over (i.e., the number of successive flower visits during which pollen export remains effective), we explored the robustness of our results and ensured that our method captures most paternal plants identified subsequently, with only 13.2% of realized mates that were not detected by our observational approach. Paternity analyses of the produced seeds further quantified performance during post-pollination selection by measuring fertilization success, which is the outcome of the filtering of pollen donors once pollen is deposited on pistils.

Interactions between mates are arguably more difficult to estimate in plants compared to animals because a single pollinator visit does not guarantee effective pollen transfer to the pollinator, its transport to a potential mate, and its deposition on a mate’s stigma. Because a simple count of the observational number of mates does not capture these complexities, we quantified two complementary measures: (i) the average number of flowers visited by pollinators at least once per mate (“flowers visited per mate” hereafter), and (ii) the average number of flowers visited on the focal plant itself. The first measure is expected to scale, for the male function, with the mean number of flowers (i.e., competitive arenas) on which a plant’s pollen competes, and for the female function, with the average quantity of pollen received from mates. The second measure is expected to scale, for the male function, with the potential for pollen export from the focal plant, and for the female function, with the maximum number of flowers that received pollen. The number of flowers visited per mate also plays a central role in our analyses to account for another plant’s characteristic: the simultaneous production of several reproductive organs associated with their modular nature. In particular, it allowed us to quantify the relative importance of reaching more mates versus reaching more of their flowers in episodes of sexual selection. We finally tested the effects of pollinator abundance on standardized metrics of sexual selection [15,34–38]. Integrating observational and genetic data in our study enabled us to disentangle the relative importance of successive episodes of sexual selection in a plant.

## Results

### Characterization of sexual selection in an insect-pollinated plant

Decomposing male reproductive success into fitness components depicting distinct episodes of sexual selection has become a classical approach to quantifying their relative importance [36,39]. We extended this approach by integrating observational data on pollinator visits with genetic data to evaluate the relative contributions of selection on access to mates (pre-pollination) versus fertilization of ovules (post-pollination). Specifically, we partitioned variance in male reproductive success into components reflecting the pollen donor’s ability to access greater numbers of observational mates, to reach more flowers per mate, to sire a greater proportion of seeds, and to access flowers with higher seed production (Fig 1A). Our results revealed that the primary driver of variance in male reproductive success was gamete fertilization ability—a fitness component typically associated with post-pollination performance (Fig 1A, S1 Table). In contrast, the observational number of sexual partners, and especially the number of flowers visited per mate, contributed only little to variance in male reproductive success (Fig 1A), which is congruent with the observation that not all flowers visited developed into fruits (63.8% ± 19.7 SD; S1 Fig).

**Fig 1:**
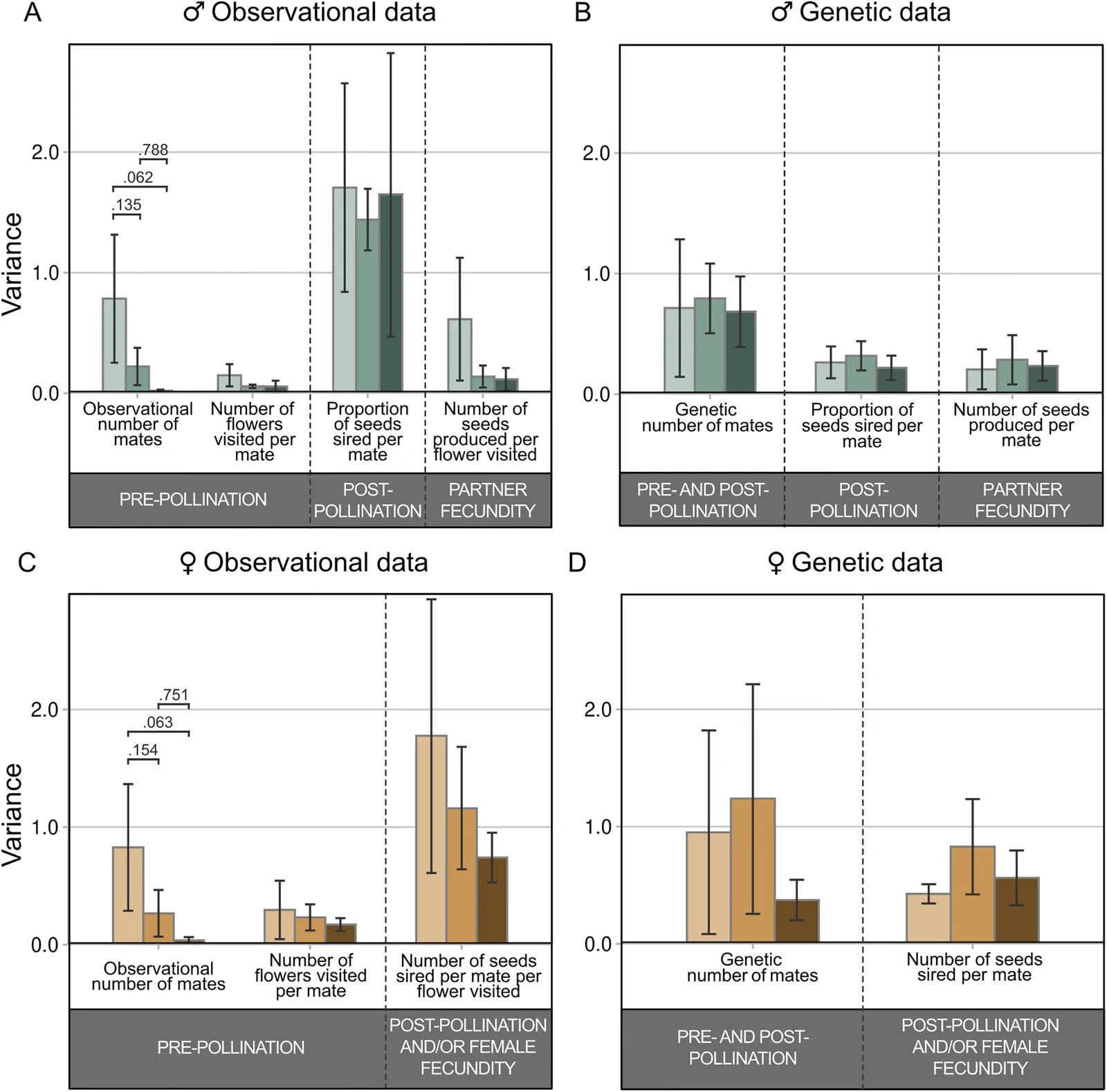
Components of sexual selection across pre- and post-pollination episodes for male and female sexual function in *B. rapa* using observational and genetic data. Variance in outcrossed reproductive success (mean ± 95% bootstrap confidence intervals) for the male (**A, B**) and female (**C, D**) sexual function is decomposed using either the combination of observational and genetic data (A, C) or solely genetic data (**B, D**) for the same experimental populations of *B. rapa* exposed to varying pollinator abundance (low: light color; medium: medium color; high: dark color). Significant overall differences in variance components between pollinator treatments, as revealed by likelihood ratio tests on nested models (S3 Table), are indicated in the figure by black connecting lines with corresponding adjusted *p*-values obtained from pairwise comparisons of treatment modalities using post-hoc Tukey’s HSD tests. The data underlying this Figure can be found at https://doi.org/10.5281/zenodo.21628183.

The alternative, traditionally used approach, which relies solely on data from genetic parentage analysis to estimate pre-pollination mate numbers [20,40–42], partitions variance into components reflecting a pollen donor’s ability to acquire genetic mates, to sire a greater proportion of seeds, and to access mates with higher fertility. In contrast to our pollinator-based approach, the decomposition based solely on genetic parentage identified the number of sexual partners, instead of gametes fertilization, as the primary driver of variance in male reproductive success (Fig 1B, S1 Table). For the female sex function, the decomposition of variance based on observational data suggests that pre-pollination fitness components such as the number of mates and the number of flowers visited per mate, contribute relatively little to the variance in reproductive success compared to the number of seeds sired by mates, a variable capturing mainly female fecundity, and therefore not pertaining to sexual selection (Fig 1C, S1 Table). In contrast, variance decomposition of female reproductive success based on genetic data alone suggested roughly equivalent contributions of all variance components (Fig 1D).

Alongside our finding that sexual selection operates primarily during the post-pollination episode of selection in *B. rapa* (Fig 1A, 1C), we found support for stronger pre-pollination sexual selection in the male compared to the female sexual function when inferred from Bateman gradients (i.e., the slope of the linear regression of offspring number on the observational number of mates, computed for individuals that were visited by a pollinator at least once; 15). Male reproductive success was positively related with the observational number of mates at both medium and high pollinator abundances, while no such relationship was found for the female sex function at any of the three tested pollinator abundances (Fig 2A, S2 Table). This translated into a sex difference in fitness benefits associated with the observational number of mates (Fig 2A, S2 Table).

**Fig 2:**
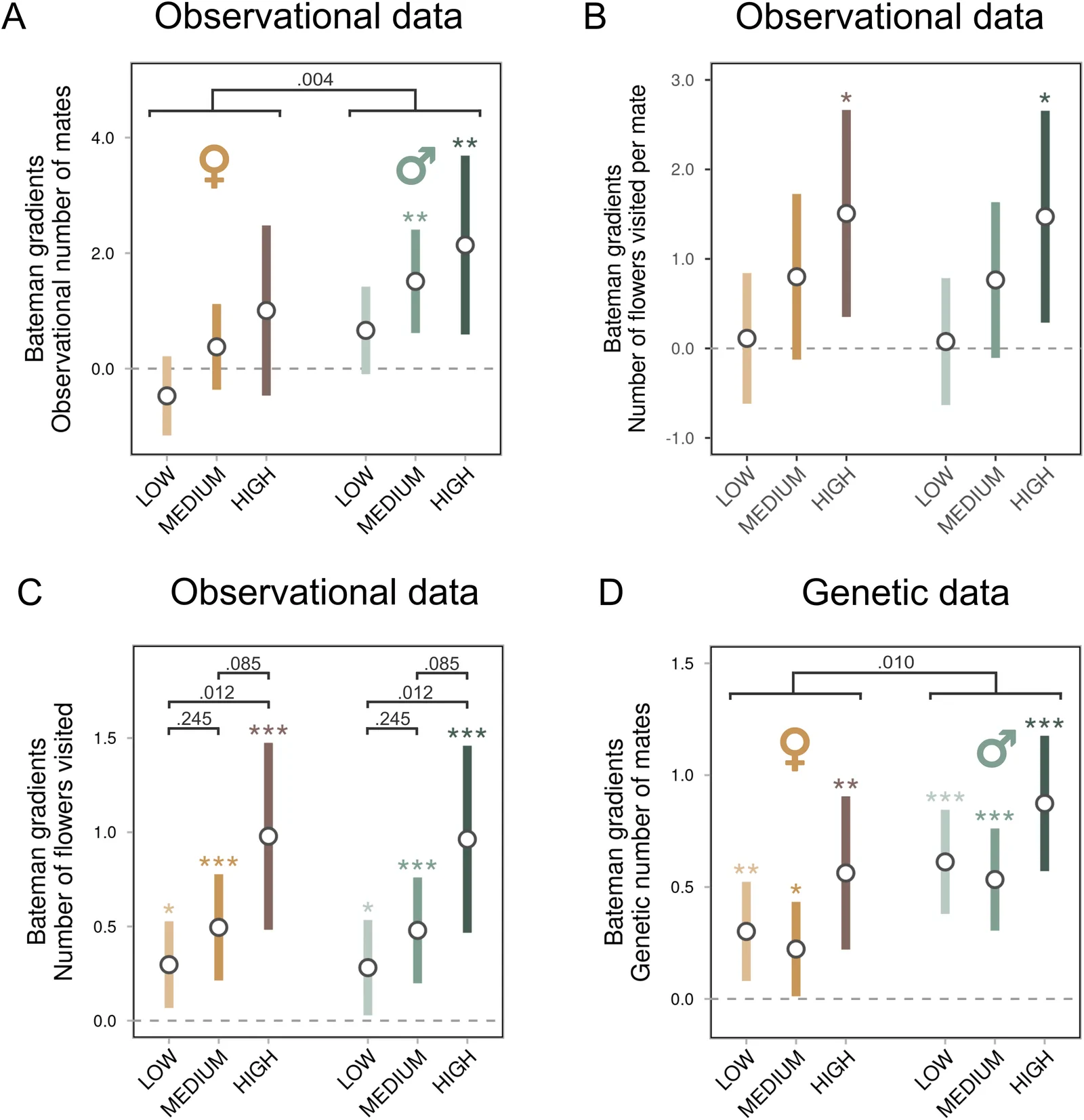
Fitness benefits of mate acquisition and pollinator visitation in the pre-pollination phase for both sexual functions across varying pollinator abundances (low: light color; medium: medium color; high: dark color). Bateman gradients are computed using total reproductive success (estimate ± 95% confidence intervals) based on the observational number of mates **(A)**, the number of flowers visited on mates **(B)**, the number of flowers visited on the plant itself **(C)**, and the genetic number of mates **(D)**. Variables related to the female function are represented in shades of orange, while those related to the male function are depicted in shades of green. Bateman gradients significantly different from zero are indicated above each bar in the corresponding color, with asterisks denoting *p*-value obtained from t-tests (• < .10, * < .05, ** < .01, *** < .001). Significant effects of treatment or sex, identified via likelihood ratio tests (S5 Table), are indicated by black lines marking pairwise comparisons performed with Tukey tests and annotated with the respective significance. The data underlying this Figure can be found at https://doi.org/10.5281/zenodo.21628183.

In contrast to sex-specific patterns identified for observational mate numbers, both male and female reproductive success increased with the number of flowers visited both per mate (i.e., a Bateman gradient computed on another component of pre-pollination performance; Fig 2B) and on the focal plant itself (Fig 2C) at higher pollinator abundance. Female reproductive success increased with larger pollen loads, but this was primarily due to the production of more selfed seeds (S2 Fig). Indeed, the effect of pollen load on female reproduction was not consistent when comparing total seed production to strictly outcrossed seed production, while our other results were robust to the use of total versus strictly outcrossed reproductive success. Bateman gradient analyses based on genetic mate numbers yielded results that contrasted with those obtained using our observational measure of mate number, as they suggested that an increased number of genetic mates was beneficial in both sexual functions (Fig 2D). Male and female reproductive success were not correlated, whereas the observational number of mating partners acquired through the male and female functions were positively correlated in the medium and high pollinator abundance treatments (S3 Table). Cross-sex effects, quantifying how increased mating success through one sex function influences reproductive success through the other function in a hermaphroditic organism, did not differ significantly from zero (S4 Table). Moreover, estimating these effects alongside Bateman gradients as classically recommended for hermaphroditic organisms [43] did not alter our main results.

### Effect of pollinator abundance on sexual selection

Our manipulation of pollinator abundance had a positive effect on the total number of pollinator visits on focal plants (S3A Fig, S5 Table). Our exhaustive monitoring of pollinator behavior revealed that those changes in pollinator visits translated into increased observational number of mates reached through pollinator movements by the male function and received by the female function (Fig 3A). Increased pollinator abundance, however, did not affect the average pollen load on the plant (S3B Fig), nor total female and male reproductive success (S3C Fig), nor the number of flowers visited both per mate (S3D Fig) and on the plant itself (S3E Fig), nor the proportion of produced selfed seeds (S3F Fig, S5 Table).

**Fig 3:**
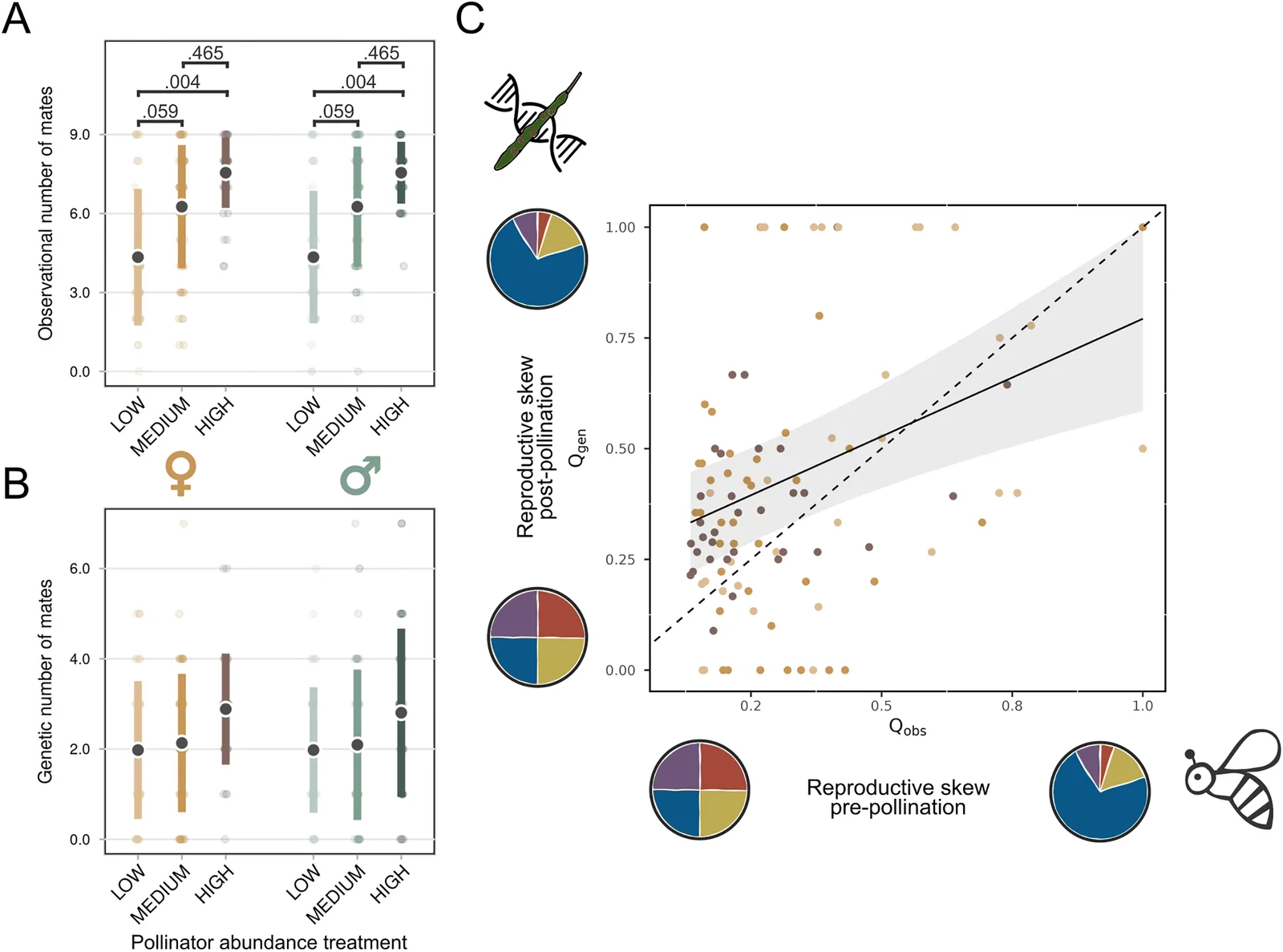
Filtering of mates during the post-pollination phase according to pollen donor diversity during the pre-pollination phase. The effect of pollinator abundance (low: light color; medium: medium color; high: dark color) on the observed number of mates during the pre-pollination episode (**A**) contrasts with the lack of a treatment effect on the number of mates that successfully fertilized seeds after the post-pollination episode **(B)**. Regression of pre-pollination reproductive skew (Q_obs_) against post-pollination reproductive skew (Q_gen_) is shown by the solid line with the associated 95% confidence interval in gray **(C)**. The relationship significantly deviated from a 1:1 line (dashed), indicating that both low and high pre-pollination skews among pollen donors were homogenized toward intermediate skew levels during the post-pollination phase. Variables related to the female function are represented in shades of orange, while those related to the male function are depicted in shades of green. Significant effects of treatment or sex, identified via likelihood ratio tests (S5 Table), are indicated by black lines marking pairwise comparisons performed with Tukey tests and annotated with the respective significance. The data underlying this Figure can be found at https://doi.org/10.5281/zenodo.21628183.

In accordance with our hypothesis that a decline in pollinator abundance should intensify the opportunity for pre-pollination sexual selection, pollinator abundance had a negative effect on the opportunity for pre-pollination sexual selection—a classical measure representing the upper limit for selection in a given episode [35]. Specifically, we found that the variance in both the observational number of sexual partners (Fig 1A, 1C) and in the number of flowers visited per mate (S4A Fig) significantly decreased for both sexes with pollinator abundance (S6 Table). In contrast, opportunities for selection calculated on variables that encompassed performance in the post-pollination phase—reproductive success and genetic mates—were not affected by pollinator abundance (S6 Table).

Our hypothesis that pollinator abundance strengthens post-pollination sexual selection in the male sex function was not supported by our results, as we did not detect any treatment effect on the variance of the proportion of seeds sired per mate (Fig 1A, S7 Table). While the observational number of mates increased significantly with pollinator abundance, the number of genetic mates, inferred after both pre- and post-pollination episodes of selection, remained unaffected by pollinator abundance (Fig 3A, 3B, S5 Table). Assuming that our inferences of observational mate numbers accurately reflect pollen deposition, this result might suggest that the most effective pollen donors at the pre-pollination phase are more discriminated against during the post-pollination phase at higher pollinator abundances. To investigate this, we tested whether the difference between reproductive skew before and after pollination—in other words, the inequity in flower contacts by pollen donors across pistils (pre-pollination) versus the inequity in the number of seeds they effectively sired (post-pollination)—varied with pollinator abundance. We found no effect of the treatment on the relationship between pre- and post-pollination reproductive skew, nor on the values of the skews themselves (Fig 3C, S8 Table). However, we observed that low pre-pollination reproductive skews (i.e., more equal flower contact among pollen donors) translated into higher post-pollination skews (i.e., less equal fertilization success), and vice versa (Fig 3C, S8 Table). This pattern suggests that when pollinator visits are uneven among pollen donors, the overrepresented donors tend to be filtered out during post-pollination processes. In contrast, when pollen donors contribute more equally in the pre-pollination phase, some are later favored during fertilization (Fig 3C).

## Discussion

While the role of sexual selection in plants has been acknowledged for decades, the first quantifications based on mate numbers in angiosperm species have only recently been conducted [19–22]. Building on these early efforts, which aligned with the vast empirical work performed in animals, our study advances the field by dissecting pre- and post-pollination episodes of selection and by integrating two key plant-specific factors, i.e., the action of pollinators as third-party players in reproduction and the simultaneous production of many reproductive organs (summary of our experimental design in Fig 4). Our results highlight that the ability to fertilize ovules in the post-pollination phase is a major component of male reproductive success in *B. rapa*. Yet, selection on mate numbers accessed in the pre-pollination phase is operating in the male but not in the female sexual function, effectively validating classical Darwinian sex-roles identified in the animal kingdom [3]. As hypothesized, pollinator abundance affected the relative importance of pre- versus post-pollination selection, which was driven by a decline in variance in the number of mates acquired during the pre-pollination phase when pollinators became more abundant. Our study underscores that relying solely on paternity analyses to estimate the number of sexual partners can lead to an overestimation of the selection component attributed to the pre-pollination phase, and to erroneous conclusions regarding the impact of ecological factors, such as pollinator abundance, on the strength and form of sexual selection.

**Fig 4:**
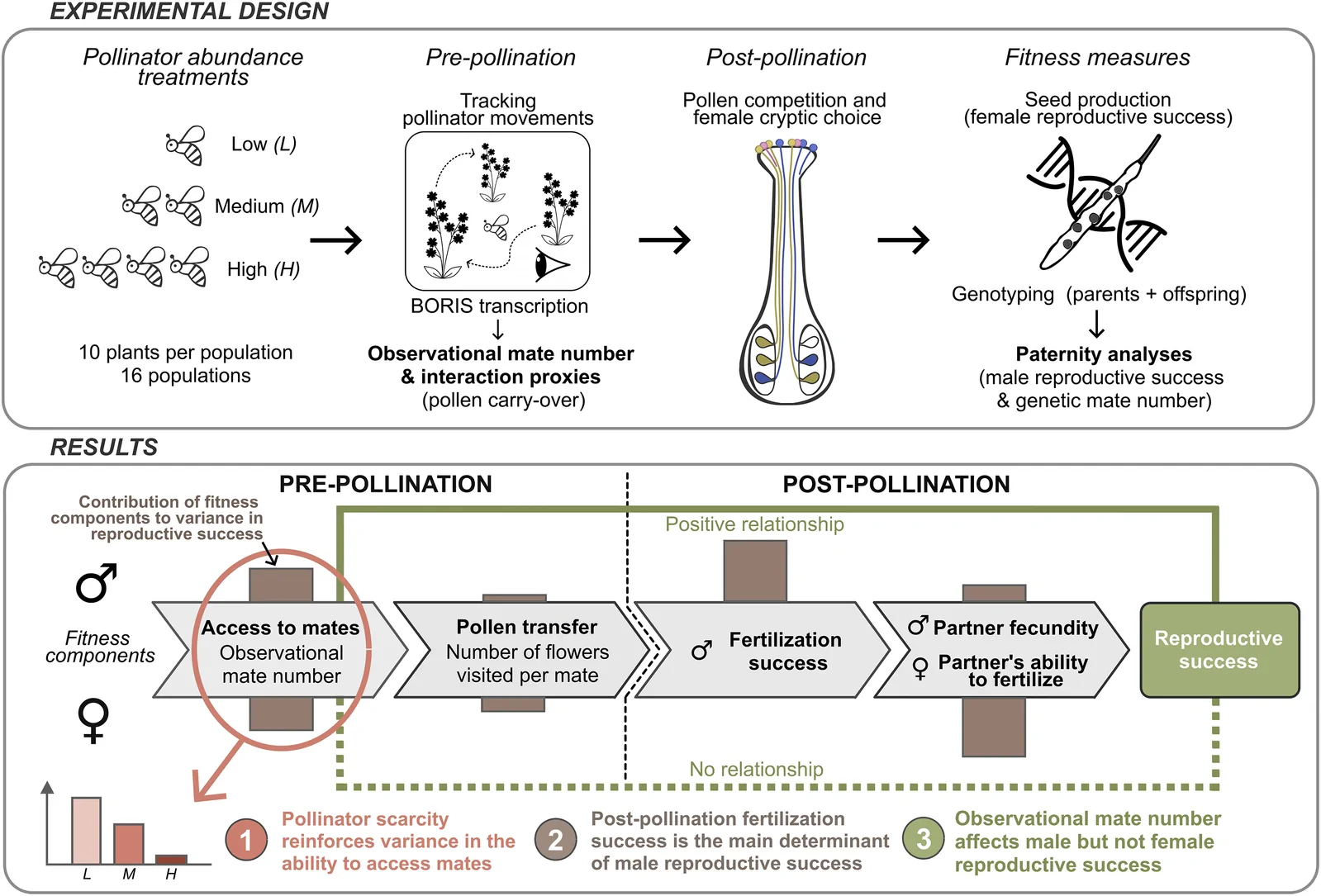
Experimental design and main results testing how pollinator abundance shapes sexual selection before and after pollination. Populations of *Brassica rapa* were exposed to low (L), medium (M), or high (H) pollinator abundance, and sexual selection metrics were quantified by following exhaustively pollinator movements and combining them with genetic paternity analyses. Pollinator scarcity increased variance in access to mates for both sexes, but post-pollination fertilization success was found to be the main determinant of male reproductive success. However, male, but not female reproductive success increased with the number of mates potentially accessed through pollinator movements in the pre-pollination phase.

### Sexual selection predominates after pollination

Our quantification of sexual selection across various phases of reproduction led to contrasting conclusions, whether the pre- and post-pollination episodes of selection were estimated independently or not. When sexual partners were counted using genetic paternity, the pollen donor’s ability to access sexual partners emerged as the primary component driving variance in male reproductive success, converging with previous findings in another angiosperm species [20]. In contrast, our approach based on the estimation of the actual number of mates obtained in the pre-pollination phase through pollinator movements, identified the pollen donor’s success at fertilizing ovules after accessing the mate, as the largest component of sexual selection for the male function (Fig 4). Sexual selection thus acted most prominently in the post-pollination phase in our experimental populations of *B. rapa*, with the pre-pollination phase gaining in importance when the likelihood of exporting pollen to mates diminished due to a decline in pollinator abundance. These different conclusions derived from genetic and observational data for estimating mating success draw caution on using the genetic number of mates as a metric to quantify sexual selection, as it confounds pre- and post-pollination episodes. Our results concur with previous recommendations in animals [31,44] to independently estimate performance in accessing mates versus fertilizing gametes when the ultimate goal is to disentangle processes of sexual selection. Yet, our approach to estimate the number of mates has its own limitations, as it relies on assumptions about the fate of pollen along flower visits, whose validity may depend on pollinator behavior. Direct tracking of pollen remains challenging as pollen marking often impedes its dispersal, and the limited availability of pollen markers constrains the number of donors that can be tracked [45]. Future studies could thus combine pollinator monitoring with pollen marking methods to refine estimates of observational mate number.

Despite the higher opportunity for sexual selection during the post-pollination episode, our study found that selection for increased access to mates during the pre-pollination phase can act on the male function of *B. rapa*. Sexual selection in the pre-pollination phase was found to operate primarily only at intermediate pollinator abundance, as this was the only condition under which the number of observed mates both positively predicted male reproductive success and contributed substantially to variance in male reproductive success compared to other selection episodes (Fig 4). Our results support a key prediction of sexual selection theory [15,17]: reproduction via the male function is more dependent on access to mates than reproduction via the female function. These sex-specific fitness returns obtained from mate acquisition align with results of a meta-analysis covering a wide taxonomic breadth in the animal kingdom [3]. Our study also extends previous work in four angiosperm clades [19–22] by providing the first sex-specific estimates of Bateman gradients in plants based on direct access of mate numbers through observation of pollinator behavior. This methodological step forward proved essential for identifying fitness gains obtained from accessing mates, because using genetic rather than observational data resulted in an overestimation of Bateman gradients. This discrepancy is expected for the male function given that genetic data often artificially re-enforces Bateman gradients, as both the number of mates and offspring are estimated through paternity outputs [44]. We also observed similar inflation for the female function, highlighting that paternity-based genetic estimates, which cap mate number at the number of viable offsprings genotyped, can lead to false positives for female Bateman gradients.

Our study empirically tests whether plant modularity alters the strength and form of pre-pollination sexual selection. We found that the number of flowers visited per mate was a minor component of sexual selection, and amounted to fitness benefits only under high pollinator abundance, where it explained a minimal proportion of the variance in reproductive success. These findings confirm that sexual selection in plants can act through access to mates rather than flowers. Indeed, the fitness benefits of broadly dispersing pollen across many flowers on each mate may be limited—especially if pollen donors that spread pollen over a greater number of flowers per mate experience progressively smaller amounts of pollen deposited on each stigma along the visit sequence. This, in turn, could weaken the probability of gamete fertilization compared to over-represented pollen donors that concentrate on fewer flowers per mate. The reported lesser importance of access to flowers compared to mates in *B. rapa* effectively suggests that the simultaneous production of sexual organs—a common plant feature that may have prevented earlier tests of pre-pollination sexual selection—does not preclude selection for mate access. However, the number of flowers visited, at least once, on a given plant or on its mates may influence the effective transfer of pollen between mates during the pre-pollination phase, thereby potentially shaping all competing pollen donors’ fertilization success in the post-pollination phase. We found that male reproductive success increased with the number of flowers visited on the donor plant, an effect that was amplified under high pollinator abundance. This pattern may reflect pre-pollination sexual selection, if producing more flowers increases the number of flowers visited and thereby enhances the quantity of pollen exported to mates. However, greater pollen export may also influence post-pollination processes (e.g., by saturating stigmas and conferring a numerical advantage). However, confirming this hypothesis requires pollen-marking techniques to quantify the relative contribution of competing pollen donors [45,46]. We also found support for a form of fecundity selection acting on the female function operating through facilitated selfing, as receiving greater pollen quantity increased the production of selfed seeds, echoing the notion of ‘gray zone’ of sexual selection proposed for such cases [47].

### Pollinator abundance affects patterns of sexual selection

Partially confirming our initial hypothesis, variation in pollinator abundance altered the relative importance of the pre- versus post-pollination episodes of sexual selection in *B. rapa*. Specifically, variance in the number of sexual partners accessed in the pre-pollination phase increased markedly under low pollinator abundance, suggesting an intensified scope for pre-pollination sexual selection under conditions of pollinator scarcity (Fig 4). Essentially, reduced pollinator abundance led to fewer pollinator visits, which consequently lowered the observational number of mates per plant. Under these low pollinator conditions—when plants exhibited greater variation in their observational number of mates—male reproductive success became increasingly influenced by access to highly fecund mates, although this effect did not reach statistical significance. These findings align with classical animal studies demonstrating that ecological conditions can strongly modulate sexual selection processes [40–42,48–50]. A similar ecological effect on sexual selection was found in a wind-pollinated plant, where selection for mate access (estimated from genetic mates) favored greater pollen dispersal distances, with different plant traits influencing dispersal at contrasting plant densities [20,51]. Selection through other components of pre-pollination sexual selection, such as pollinator visits, is also commonly affected by ecological factors [52,53].

Our results that the opportunity for sexual selection focusing on mate acquisition during the pre-pollination phase increases under reduced pollinator abundance raises the question whether ongoing pollinator declines could intensify this component of sexual selection, with potential consequences for plant populations. Theory posits that the expression of sexually selected traits is condition-dependent, meaning that it is determined by the pool of resources an individual can allocate towards different fitness routes, including the acquisition of mates and the fertilization of their gametes. On the assumption that this pool of acquired resources is a highly polygenic trait defining an individual’s overall vigor and health, selection on condition-dependent sexually selected traits is predicted to facilitate genome-wide purging of deleterious alleles [54]. This condition-dependent aspect of sexual selection has been well documented in animals [55–57], with possible positive effects on population dynamics [58,59] and the purging of deleterious mutations [60–62]. Because our experiment relied on caged bumblebees, it may however not fully capture natural foraging dynamics. Methodologies enabling large-scale tracking of pollinators, such as radio tracking [63,64], will be needed to determine whether sexual selection operates similarly in natural populations, and whether it could generate positive effects on population dynamics under pollinator decline. Addressing the interaction between pollinator declines and the postulated demographic effects of sexual selection also requires quantifying genetic correlations between sexually selected traits under low pollinator abundance and other components of plant fitness under natural pollination.

Our initial hypothesis that pollen donors would be increasingly discriminated against during the post-pollination phase under higher pollinator abundance was not supported by our results. Although the absolute number of mates received by the female function during the pre-pollination phase increased with pollinator abundance, the quantity of pollen deposited on stigmas did not vary significantly. Surprisingly, this rise in polyandry did not lead to a corresponding increase in the number of genetic mates contributing to seed paternity, a result that may indicate a treatment-dependent paternity skew. To investigate whether, consistently with this discrepancy, a larger proportion of pollen donors were filtered out during the post-pollination phase at higher pollinator abundance, we examined whether inequalities in pollen receipt among donors were amplified after pollination. Our results revealed a rebalancing of pollen donor contributions during the post-pollination phase, which was not affected by pollinator abundance. Specifically, when pollen contributions were highly skewed during the pre-pollination phase, initially overrepresented donors tended to be filtered out during post-pollination processes. In contrast, when pollen was more evenly distributed among donors beforehand, some were subsequently favored after pollination. These findings are consistent with our variance decomposition results, underscoring the role of post-pollination sexual selection in reshaping male reproductive skew between pre- and post-pollination phases. Future research on sexual selection should address whether these post-pollination filtering processes are strictly attributable to pollen competition or whether a form of cryptic female choice is also involved, as our data cannot tease them apart [13,30].

By accounting for key characteristics of plant reproduction, we quantified episodes of pre- and post-pollination sexual selection independently in an insect-pollinated angiosperm. This novel approach allowed us to uncover substantial complexities in sexual selection processes that would have remained hidden using genetic data alone. Notably, our findings emphasize that the relative contributions of pre- and post-pollination episodes of selection are not a fixed attribute of a species but are influenced by ecological conditions, particularly pollinator abundance in insect-pollinated plants. We advocate that testing sexual selection theory across anisogamous sexually reproducing clades using approaches directly connected to theory and tailored to the unique characteristics of each clade could foster the development of a unified and comprehensive framework for sexual selection applicable to all sexual organisms.

## Materials and methods

### Study system and biological materials

Our experimental populations were set up using *Brassica rapa* as the plant species and *Bombus terrestris* as its pollinator. *B. rapa* is a hermaphroditic, herbaceous, insect-pollinated plant species belonging to the Brassicaceae family. It is typically self-incompatible owing to a genetic self-incompatibility locus common to all members of the *Brassica* genus [65,66]. However, this genetic self-incompatibility mechanism is not complete, and variable outcrossing rates are commonly observed in *B. rapa*, including in the line used in this study where selfing has been shown to evolve during experimental evolution [27]. For this study, we used the commercial line *B. rapa* Wisconsin Fast Plants (WFP; Carolina Biological Supply Company, Burlington, North Carolina, USA), a line artificially selected for a short generation time (~8 weeks) while maintaining substantial phenotypic and genetic variation [67]. The experimental populations in our study were composed of plants originating from five experimental populations of *B. rapa* WFP that had undergone three to four generations of selection under strong polygamy. Specifically, seeds were harvested from 85 parental plants in these populations, where each plant had three flowers pollinated with pollen mixes from 12 anthers collected per plant across the population. After each generation, seeds from the three pollinated flowers were bulked to establish the next generation. The seeds used in this study were mixed from the populations of the third and fourth generations of these populations and are hereafter referred to as the ‘source population’. The pollinator, *B. terrestris*, is a social insect that forms colonies and feeds on a wide variety of plant species [68]. This species is an efficient pollinator of *B. rapa* [25,32,33], making it well-suited for our experimental design.

### Experimental design

We established 16 experimental populations of *our source population of B. rapa*, each exposed to one of three levels of pollinator abundance: low, medium, or high. Seeds from our source population were sown in greenhouses at an experimental platform in Montpellier, France, from December 2021 to January 2022. To ensure sufficient plant availability, seeds were sown in three temporal blocks staggered by 1–2 weeks, yielding a total of 210 plants (~70 per block). Plants were grown under standardized conditions, with continuous light at 25 °C, in separate 0.7 L pots filled with a sterilized soil mixture. Each experimental population consisted of 10 flowering *B. rapa* plants randomly chosen among a given temporal block, standardizing plant age to 3–4 weeks at the time of pollination. The selected plants were placed in a mesh cage to confine pollinators during the experiment. Pollinator abundance treatments consisted of exposing the plants to one bumblebee (six replicates), two bumblebees (six replicates), or four bumblebees (four replicates). Prior to each pollination session, pollinators were marked with distinct paint colors to enable individual tracking. Observers recorded verbally the movements of their assigned pollinator in real time, specifying the plant, stem, and flower position visited (by counting flowers downward from the top), for ~30 min per experimental population, excluding from this counting any initial phase of complete absence of pollinator movements. A flower was only reported as visited if the pollinator came into contact with the sexual organs. To facilitate the recording of pollinator positions, each plant stem had been marked with distinct paint colors, and all open flowers with red paint. Because flower number is known to influence pollinator attractiveness [69], we counted the total number of open flowers on each plant. Flower numbers did not differ between treatments according to likelihood ratio tests comparing models with and without pollinator treatment as a fixed effect and experimental population as a random effect (low: 15.6 ± 1.02 SE; medium: 14.3 ± 0.95 SE; high: 14.4 ± 1.08 SE; $X^{\text{2}}=\text{0}.\text{1}\text{8}$, P=0.91, df=1, N=160). Pollinators were obtained from commercial *B. terrestris* hives (Biobest, Orange, France). To account for potential variability in hive activity, one hive was randomly selected for each pollination session from the four hives purchased with the same representation of each hive across treatments. Across all 16 experimental populations, a total of 160 parental plants were phenotyped and subjected to pollinator observation. After the observation sessions, plants were transferred to an insect-proof greenhouse to ensure controlled fructification. Seeds were subsequently collected for paternity analysis.

### Exhaustive recording of pollinators visits and estimation of observational mate numbers

We transcribed our real-time observational data of pollinator movements recorded at the flower scale for each plant using the BORIS software [70]. This procedure resulted in a dataset documenting, for each visit, the time of the pollinator arrival on a given plant, specifying the plant and flower identity. The primary goal of the pollinator real-time observation was to infer the number of mates obtained by plants during the pre-pollination phase via transport of pollen, referred to in this study as ‘observational number of mates’. This observational mate number was derived solely from the pollinator visit dataset. For the male function, it represents the maximum number of distinct plants to which transfer of a focal plant’s pollen was made possible by pollinator visits. For the female function, it corresponds to the maximum number of distinct pollen donors implied by pollinator visits on the flowers of the focal plants. This estimation of the observational number of mates is conceptually equivalent to the number of copulatory mates traditionally measured in studies of sexual selection in animals [1,34,37,43,44].

While our experiment provides exhaustive data on pollinator movements, we did not track the fate of pollen on pollinator bodies or its transfer among plants. This decision reflects the considerable challenges associated with tracking transport of pollen. Available methods for marking pollen have significant limitations, including: (i) alterations of pollen properties that may hamper its dispersal capabilities [71]; (ii) potential reductions in pollen viability due to chemical treatments, which would bias the decomposition of selective processes occurring in the pre-pollination and post-pollination episodes of selection [45]; and (iii) the limited number of distinguishable colors for marking pollen, which complicates assessments of mate numbers for multiple competing plants [45]. To infer observational mate numbers from pollinator visitation data, we formulated hypotheses regarding the number of successive flower visits during which pollen export remains effective, referred to as pollen carry-over [72,73]. Our primary analysis was based on a pollen carry-over of 10 flowers consistent with empirical estimates in a closely related species [74]. To evaluate the robustness of our conclusions, we conducted a sensitivity analysis across a range of pollen carry-over scenarios. Specifically, we tested whether results remained significant for incremental changes in assumed carry-over, and reported the range of values for which our results remained consistent (see Supplementary Tables in Supporting information). The same procedure was applied for the variable quantifying the number of flowers visited per mate, as its computation also requires pollen carry-over data (see below). To validate our methodology, we confirmed that under the highest pollen carry-over, we recovered the majority (86.2%) of paternal plants identified as fathers through paternity analysis among the recorded observational mates.

### Estimation of the intensity of interactions between mates and pollen transfer

Our computation of the observational mate number does not necessarily reflect whether interactions between sexual partners are ultimately effective. For successful pollen transfer between plants—analogous to mating in animals—pollen must be transferred to the pollinator body, transported, and deposited onto the stigma of the recipient plant [10]. The actual number of mates accessed may therefore depend on the number of pollinator contacts on the mate, which may reflect actual pollen transfer. We considered that the number of flowers visited on the mates (averaged across all mates) and on the focal plant may be a reliable indicator of the effectiveness of pollen transfer between mates as it may reflect larger quantities of pollen exchange between mates. In addition to quantifying the intensity of pollinator visits on mates and the focal plant’s attractiveness, we estimated the quantity of pollen deposited on pistils, referred to as pollen loads [75]. Specifically, we collected two to three stigmas per plant 5 days after the pollination session and once fructification had started, and assessed the number of pollen grains deposited, following the protocol described in the S1 Text [76,77].

### Genotype acquisition, paternity analyses, and estimation of the genetic number of mates

When plants had completed their life-cycle, we counted all produced fruits, harvested them, and both counted all seeds for later assessment of the variance in reproductive success via the female function. On average, seed number was assessed in 6.52 (SD ± 4.37) fruits per maternal plant, yielding a total of 959 counted fruits. We assessed germination rate by sampling seeds proportionally to the number of seeds produced (mean ± SD: 22.9 ± 27.4 per maternal plant), which were sown in Petri dishes filled with 40 mL of 10 g/L agar in sterile water (photoperiod: 16:8, temperature: 20–23 °C) for 1 week. For each maternal plant that produced seeds (137 in total), 10 seedlings were selected for subsequent DNA extraction and genotyping for paternity analysis, except for 25 individuals that produced fewer than 10 viable seeds and for four individuals that failed to produce any viable seeds. DNA extraction, single-step PCR assays, and capillary electrophoresis for both adults and seedlings were performed as described in the S2 Text. In a nutshell, samples were subjected to a tissue homogenizer, followed by DNA extraction using magnetic beads and an automated purification workstation. Single-step multiplex PCR assays were then used to amplify eight nuclear microsatellites, and electrophoresis was performed with a size standard (S9 Table). Alleles at the eight loci were assigned using GeneMapper (Thermo Fisher Scientific, Waltham, USA) for each parent and offspring, resulting in genotypes for 160 parents and 1,193 seedlings.

Paternity analyses were conducted using CERVUS 3.0.7 [78,79]. This software performs categorical paternity assignments by simulating a large number of reproductive events with simulated parents of known genotypes, sampled accordingly to estimated allelic frequencies in the study population. This simulated dataset with known paternity allows for the calculation of likelihood score distributions and determination of a critical value for assigning the true father to a given seed at a specified confidence interval. Paternity is assigned to a given individual based on likelihood ratios, comparing the likelihood of observing the offspring genotype assuming the individual is the true father, to the likelihood assuming a random male is the father. We used an 80% confidence level (relaxed criterion) to retain the most-likely father, allowing for up to three minimum typed loci and 1% genotyping error (estimated based on mismatches between mothers and offspring, which averaged 0.084% across all eight markers). The mean nonexclusion probability was 0.098 across the 16 sessions, with a range from 0.050 to 0.15. The assignment procedure successfully assigned paternity to 1,099 seedlings (mean ± SD: 6.87 ± 3.97 per maternal plant). Female and male genetic number of mates were computed as the number of sexual partners with whom a given plant shared at least one seed following fertilization. Thus, this variable captures not only access to mating partners determined during pre-pollination selection, but is also affected by fertilization success, which is subject to post-pollination selection. Only a small proportion of the genotyped seeds (7.73%; 85 seeds out of 1,099) exhibited genotypes that were not compatible with our observational data of pollinator visits under an infinite carry-over model, confirming the validity of our approach for inferring observational mate numbers based on pollinator observations.

### Estimation of sex-specific reproductive successes

For both sexes, we calculated both total reproductive success and strictly outcrossed reproductive success. Total female reproductive success was quantified as the total number of seeds produced, weighted by the estimated seed germination rate for each female. Outcrossed female reproductive success was estimated as the proportion of seeds produced with another plant (assessed by genotyping seeds) multiplied by total female reproductive success. Male reproductive success was quantified as the sum, across all mothers, of the product of (i) the proportion of seeds sired by the focal male on each mother by (ii) the mother’s reproductive success. Female and male reproductive successes were calculated by either excluding or including the selfed seeds. We also computed reproductive successes without weighting seed production by germination rates to avoid artificial correlation between female reproductive success and the number of genetic partners that may emerge because the genetic number of mates could solely be assessed on germinated seeds for the female function. As most of our results were robust to the inclusion or exclusion of selfed seeds, we presented our results considering total reproductive success, but any lack of robustness when using outcrossed reproductive success is shown in Supporting information and discussed. However, several additional experiments have found that selfed seeds experience strong inbreeding depression, therefore suggesting that the outcrossed fraction of seeds contributes more to reproductive success (JT. pers. communication).

### Statistical analysis

All analyses were conducted in R version 4.5.2 [80]. The analysis scripts and the list of required R packages are available at https://doi.org/10.5281/zenodo.21628183*.*

### Effect of pollinator abundance on pollinator visits and reproductive performance

To test whether pollinator abundance influenced pollinator visits received by individual plants, we first compared mixed-effects models explaining the total number of visits received per plant, with and without treatment as a fixed effect, using likelihood ratio tests. The same procedure was applied to evaluate the effect of pollinator treatment on pollen loads, plant reproductive success, both observational and genetic number of mates, and the number of flowers visited on the mates and on the plant itself. Specifically, we compared nested mixed-effects models explaining sequentially one of these variables and including or not the sex by treatment interaction. Post-hoc Tukey tests were conducted to test for pairwise differences between levels of pollinator abundance and/or sexual functions when significant effects of treatment, sex, or their interaction were observed. All models throughout the statistical analysis included random effects for experimental populations and nested random effects for individuals within session, the latter because a plant could be used in different sex roles in different sessions, or because several measures were taken per plant. Each response variable was modeled using the most appropriate error distribution, as reported in S5 Table. When appropriate, statistical analyses were performed with and without individuals that failed to reproduce.

### Opportunity for (sexual) selection based on pollinator abundance

We estimated two classical metrics for quantifying the upper limits of selection and sexual selection: the opportunity for selection (*I*) classically defined as the variance in reproductive success divided by the square of the mean reproductive success, while the opportunity for sexual selection, (*I*_*S*_) was defined as the variance in mate number divided by the square of the mean mate number [40]. We estimated *I*_*S*_ using both observational or genetic mate numbers, as well as on the number of flowers visited on mates and on the plant itself. All computed metrics of selection and sexual selection were calculated for each independent experimental population and each sexual function. Following established recommendations [39,81,82], variances in reproductive success were corrected for the expected binomial sampling error for both sexes. Specifically, variance in male reproductive success was corrected for the binomial sampling of focal offspring, while variance in female reproductive success was corrected for the binomial sampling of outcrossed versus selfed seeds. The effects of treatment, sex, and their interaction on opportunities for selection and sexual selection were tested using likelihood ratio tests, and differences between pollinator treatment modalities were estimated using *t*-tests.

### Quantification of Bateman’s gradients

Bateman gradients classically quantify fitness benefits of increased access to sexual partners [15,37]. Building on classical methods in animals, we estimated Bateman gradients as the slope of the regression of reproductive success on either the number of mates (observational or genetic) or the number of flowers visited on mates and on the plant itself. Individuals that did not reproduce (and thus had zero sexual partners) through either sexual function were removed from these analyses to avoid inflation of the estimated slope. Prior to the estimation of Bateman gradients, we relativized both response and explanatory variables by dividing each value by the mean within each sexual function and experimental population [44,83]. To test whether Bateman gradients differed between sex and pollinator abundance treatments, we built linear mixed models explaining the relativized reproductive success as a function of one of the relativized explanatory variables described above, by pollinator abundance treatment, sex, and their interaction. Specifically, we used likelihood ratio tests to compare models including or not the three-way interaction term. When a significant three-way interaction was detected, we extracted slope estimates and significance levels for each combination of pollinator treatment and sexual function, by modifying contrasts in the model. When the three-way interaction was not significant, we assumed that pollinator treatment effects were consistent between sexual functions and tested for two-way interactions between the explanatory variable and either pollinator treatment or sex. Bateman gradients were calculated using reproductive success estimates that did not account for germination rates. Including germination rates could lead to false detection of positive Bateman gradients in females because the number of genetic sexual partners, by being counted on germinated seedlings, is necessarily positively related to germination rates. Results robustness were also checked by including germination rate. Our quantification of sexual selection was primarily carried out by the traditionally more established metrics in the field, i.e., opportunities of selection and Bateman gradients [15,35]. For the sake of completeness, we also report results obtained for Jones indexes, a metric quantifying the maximal strength of sexual selection as the product of the opportunity for sexual selection (*I*_*S*_*)* and the square root of the Bateman gradient [34]. This metric was recently argued to better capture sexual selection in the pre-mating phase in animals [38], and results obtained in our study were congruent with our conclusions based on the other traditional metrics (S10 Table). Finally, we calculated partial Bateman gradients [84,85] with flower number included as a covariate. As accounting for flower number did not alter our results, we report only the results from the classic Bateman gradients.

Both sexual functions are jointly expressed in hermaphrodites; thus, resource allocation to acquiring mates in one sexual function could influence the reproductive success of the other [44,81]. Additionally, performance in one sexual function may correlate with performance in the other because individuals with greater resource acquisition can allocate more resources to both sex functions. To describe the reproductive biology of our hermaphroditic species, we first quantified correlations between the two sexual functions in terms of reproductive success, observational and genetic sexual partners. As the production of selfed seeds is necessarily correlated for the male and female sexual functions, this analysis was restricted to the outcrossed fraction of reproductive success. Mixed-effect models were used to regress male-function values on female-function values, accounting for the random effects for experimental populations and nested random effects for individuals within sessions. To complement our primary analysis of Bateman gradients, we estimated cross-sex effects, which capture whether mate numbers obtained via the male function affects reproductive success via the female function, and vice versa [81]. Cross sex-effects were excluded from the main analysis as they were neither statistically significant individually, nor in interaction with sex or treatment (S4 Table). Furthermore, their inclusion did not alter the primary results of the analysis.

### Decomposition of variance in male and female reproductive success

Variance in male and female reproductive success was decomposed into its components, to identify the factors contributing most to the total variance and to investigate how pollinator abundance treatments affected their relative contributions. This variance decomposition was performed using classical methods based on genetic estimates of mate numbers [39], and by adapting this methodology to data on pollinator observation. This allowed us to compare results from genetic mates estimates with those derived strictly from pollinator movements in the pre-pollination episode. Detailed equations for the sex-specific variance decomposition methods, for genetic and observational data, are provided in the S3 Text. For this specific analysis, we omitted variance in selfed offspring as the main goal was to decompose episodes of sexual selection. In summary, variance in male outcrossed reproductive success was first decomposed into components including the number of genetic partners, the proportion of seeds fertilized per partner, the partner fecundity, and covariances among these factors (S5 Fig). Using observational data, variance in outcrossed male reproductive success was decomposed into the number of observational partners accessed, the number of flowers on which pollinators deposited pollen per mate, the number of seeds produced by mate per flower accessed, the proportion of seeds fertilized within these flowers, and all associated covariances (S5 Fig). For the female function, variance in outcrossed reproductive success was decomposed into the number of genetic mates and the number of offspring produced with each mate, along with the covariance between these two components. Using observational data, variance in outcrossed female reproductive success was also decomposed into the number of mates whose pollen landed on the pollen recipient’s stigma, the number of flowers accessed by pollen of a given mate, and the number of seeds produced by flower accessed. All components except for those describing seed production in females, which pertains to female fecundity, reflect different components of sexual selection. All variance components were computed at the level of the experimental population. Individuals that did not reproduce were included in the computation of the variance in the number of mates. We tested whether these components differed with pollinator abundance, following methods described above for the metrics of opportunities of selection. To test for qualitative differences in the contributions of variance components, we compared nested mixed models with a random effect of the experimental population explaining the variance attributed to each component, with and without the identity of the variance components, using likelihood ratio tests. When variance differences between components were detected, Tukey tests were applied to identify the largest contributing component of reproductive success variance.

### Inference of discrimination of pollen donors during the post-pollination phase

To test for discrimination of pollen donors during the post-pollination phase, we compared the diversity of observational mates to the diversity of genetic mates. However, the number of mates, as usually considered in Bateman gradients, is poorly suited for such comparisons. The number of genetic mates of a focal plant in the female role was derived from a lower number of observations (the number of genotyped seeds) than the number of observational mates (derived from observations of pollinator visits). So the inferred number of genetic mates is expected to be lower than the number of observational mates, with a reduction factor depending on sample size and on the distribution, among pollen donors, of number of visits on the focal plant, as well as on genetic sample size. To avoid this inherent bias, we used a measure of reproductive skew, defined as the frequency of pairs of distinct visits (or of pairs of parentages of distinct seeds) that involve a single mate, to quantify the diversity of mates: the higher the frequency, the higher the skew. The reduction in genotyped sample size does not per se bias the comparison between diversities of observational and retained mates after fertilization. A well-known measure of reproductive skew, Morisita’s index [82,86], is actually identical, up to a multiplicative constant (the number of potential mates), to this frequency (hereafter *Q*).

We thus fitted the frequency *Q*_*gen*_ among genotyped seeds to the frequency *Q*_*obs*_ among observed visits, by linear mixed-effect models. We allowed heterocedasticity of residuals, which is here expected by design, by fitting the residual variance in terms of the number of genotyped seeds from which each response value *Q*_*gen*_ was determined. These heteroscedastic models were adjusted with the spaMM R package [83]. The full model allowed for an interaction between *Q*_*obs*_ and pollinator abundance.

In the absence of any discrimination of pollen donors during the post-pollination phase (through male-male competition and/or cryptic female choice), the fit should be consistent with a 1:1 relationship between *Qgen* and *Qobs*. Equalization of pollen donors contributions during the post-pollination phase will increase *Qgen* above *Qobs*, and conversely it will reduce *Qgen* below *Qobs* if the mother plant selects specific mates or if competition among pollen donors favors some of them. We compared more or less constrained models, including a model where the regression line was forced through the point on the 1:1 line corresponding to the highest observed value of *Qobs*. If this fit is not rejected by a test in comparison with the unconstrained model, then it cannot be rejected that the *Qobs* predicted by the fit are always above or always below *Qgen*, depending on whether the fitted regression slope of the constrained model is lower or higher than 1, respectively.

## Supporting information

S1 Text Pollen loads estimation. (PDF)

S2 Text DNA extraction, PCR and electrophoresis. (PDF)

S3 Text Variance decomposition analyses. (PDF)

S1 Fig Distribution of the proportion of flowers visited per mate that give fruits with seeds. (PDF)

S2 Fig Effects of the pollinator abundance treatment on the Bateman gradients on pollen loads for both reproductive successes. (PDF)

S3 Fig Effect of pollinator abundance on pollinator visitation, pollen loads, and plant reproductive success. (PDF)

S4 Fig Effects of pollinator abundance on the opportunities for selection. (PDF)

S5 Fig Effect of pollinator abundance treatment on variance components of outcrossed reproductive success. (PDF)

S1 Table Results of likelihood ratio tests testing for the main component of reproductive success for both sexual functions according to the data type employed for variance decomposition. (PDF)

S2 Table Effects of pollinator abundance treatment, sex (when appropriate), and their interaction on estimated Bateman gradients. (PDF)

S3 Table Relationships between male and female fitness components assessed for the three tested pollinator abundances separately. (PDF)

S4 Table Results of likelihood ratio tests testing for the cross-sex effects for the Bateman gradients using either the genetic number of mates or the observational number of mates, for each sexual function. (PDF)

S5 Table Results of likelihood ratio tests testing for the effects of pollinator abundance treatment, sex, and their interaction, on the measured variables. (PDF)

S6 Table Results of likelihood ratio tests on the effects of sex, treatment, and their interaction on the opportunities for selection (*I*) and the opportunities for sexual selection (*Is*). (PDF)

S7 Table Results of likelihood ratio tests testing for the effect of pollinator abundance treatment on the variance components of the female and male reproductive successes. (PDF)

S8 Table Results of likelihood ratio tests for model comparison on the relationship between genetic and observational number of mates using reproductive skew measures (*Q*_*gen*_ versus *Q*_*obs*_). (PDF)

S9 Table Properties of the eight nuclear microsatellites used for individual allelic characterization and paternity analysis. (PDF)

S10 Table Results of likelihood ratio tests testing for the effects of pollinator abundance treatment, sex, and their interaction, on Jones indexes. (PDF)
