# Supplementary material for: Pollinator abundance shapes sexual selection in an angiosperm
Source: PLoS Biol. 2026 Oct 27;24(10):e3003579. doi: 10.1371/journal.pbio.3003579 (PMC13712744; doi:10.1371/journal.pbio.3003579)
Supplement: Supplementary file 1 [file pbio.3003579.s001.pdf]

Supplementary Text S1. Pollen loads estimation.

Two to three stigmas were collected on each individual five days after pollination. Our preliminary tests on *B. rapa* Fast Plants showed that harvesting stigmas five days after pollination did not affect seed production. In the current study, the number of seeds produced did not differ between flowers with and without harvested stigmas, as shown by comparing models explaining seed set with or without a variable describing the stigma status (harvest or intact) using likelihood ratio tests ($X^2 = 1.43$, $df = 1$, $N = 590$, $P = .231$). This test was performed on a dataset including 590 fruits (i.e. 214 with removed stigmas and 376 with intact stigmas). The observation session and individual plants nested within sessions were treated as random effects in this analysis. Once collected, stigmas were stored in separate eppendorfs containing FAA (ethanol 70%, formaldehyde 35% and acetic acid, 8:1:1), which allows long time conservation of pistil tissues while preserving the integrity of pollen grains before counts. Stigmas were then immersed in a bath of 10µL of sodium hydroxide (NaOH 4 $mol.L^{-1}$) for at least 3 hours to soften tissues, and then washed for one hour in a bath of 10µL of water. Stigmas were then mounted on a microscope slide with Alexander solution to color pollen grains [1], gently crushed to place tissue in the same plane, and observed at x10 magnification (Olympus CX43; Olympus Corporation, Tokyo, Japan). We took pictures of the whole stigmatic surface using a camera adaptator (Olympus U-CMAD3; Olympus Corporation, Tokyo, Japan) for later counts with Image J [2]. To account for potential loss of pollen grains during tissue preparation, we also counted for each sample the remaining pollen grains in the two baths of sodium hydroxide and water by depositing the solutions on Mallassez cells and counting pollen grain directly (2.5µL for each solution). The number of pollen grains, i.e. pollen load, on each stigma was finally obtained by summing counts on all pictures as well as pollen grains counted in the solutions of sodium hydroxide and water.
