# Supplementary material for: Pollinator abundance shapes sexual selection in an angiosperm
Source: PLoS Biol. 2026 Oct 27;24(10):e3003579. doi: 10.1371/journal.pbio.3003579 (PMC13712744; doi:10.1371/journal.pbio.3003579)
Supplement: Supplementary file 2 [file pbio.3003579.s002.pdf]

Supplementary Text S2. DNA extraction, PCR and electrophoresis.

Total genomic DNA from adults was extracted from approximately 12 mg of dried leaves, while offspring DNA was directly extracted from young seedlings. To obtain the seedlings, seeds were sown on petri dishes containing agar in sterile water (10 $mg \cdot L^{-1}$) and incubated for one week under a photoperiod of 16:8 (light:dark) at temperatures ranging from 20 to 23°C.

DNA extraction for both adults and offspring was performed using the NucleoMag Plant kit for DNA purification (Macherey-Nagel Inc., Allentown, USA), following the manufacturer's instructions. A summary of the extraction process is provided below. Biological samples were initially crushed using a tissue homogenizer with tungsten beads (Qiagen, Hilden, Germany). The samples were then incubated in a thermolyser with RNase and a lysis buffer. After centrifugation, the clear lysates were transferred to deep-well plates (Thermo Fisher Scientific, Waltham, USA) containing a buffer and magnetic beads. DNA purification was automated using a KingFisher® workstation (Thermo Fisher Scientific, Waltham, USA), where successive washes with either kit buffers or 80% ethanol yielded purified DNA, which was eluted in the kit's elution buffer.

Single-step multiplex PCR assays were performed on each DNA extract to amplify eight nuclear microsatellites (Table S8), using Applied Biosystems™ Master Mix (Thermo Fisher Scientific, Waltham, USA). The PCR cycling program included the following steps: (i) initial denaturation at 95°C for 15 minutes; (ii) amplification for 30 cycles consisting of 95°C for 30 seconds, 58°C (annealing) for 1 minute and 30 seconds, and 72°C for 1 minute; (iii) final extension at 60°C for 30 minutes.

PCR products, diluted 1:120 in distilled water, were analyzed via capillary electrophoresis (Applied Biosystems® 3130 Genetic Analyzer). GeneScan™ 500 LIZ™ (Thermo Fisher Scientific, Waltham, USA) was used as the size standard for allelic profile characterization, which was performed using GeneMapper® software (Thermo Fisher Scientific, Waltham, USA).
