# Supplementary material for: Pollinator abundance shapes sexual selection in an angiosperm
Source: PLoS Biol. 2026 Oct 27;24(10):e3003579. doi: 10.1371/journal.pbio.3003579 (PMC13712744; doi:10.1371/journal.pbio.3003579)
Supplement: Supplementary file 3 [file pbio.3003579.s003.pdf]

Supplementary Text S3. Variance decomposition analyses.

Reproductive success was decomposed into its components by extending established methodologies adapted to *Brassica rapa* [3–5] using both the classical metric of the genetic number of mates and the observational number of mates. The latter is employed for the first time in decomposition of plant reproductive success, prompting the development of a novel analytical framework to assess the effects of different components contributing to variance in plant reproductive success. The objectives of this analysis were to: (i) identify the components contributing most to variance in reproductive success for each sexual function, (ii) determine whether the relative contributions of these components varied between pollinator abundance treatments, and (iii) compare results obtained using genetic versus observational mate numbers. To achieve these goals, we first decomposed variance in reproductive success using estimates of mate numbers based on (i) mates sharing at least one produced seed (genetic number of mates, encompassing both pre- and post-pollination processes) or (ii) mates acquired through pollinator movements, which includes only pre-pollination processes. Depending on the proxy used (genetic or observational number of mates), we further decomposed variance into its successive components, as described in detail below.

The analysis focused exclusively on outcrossed reproductive success, as selfing can occur independently of pollinator visits, precluding its decomposition using observational mate numbers. As variance in reproductive success was decomposed at the scale of the population and per sexual function, one population was excluded from the dataset because only two individuals in this population reproduced via outcrossing, precluding us to estimate variance component. Additionally, for variance decomposition using the observational number of mates, we restricted the analysis to outcrossed seeds that were compatible with our data on observed pollinator visits, thereby excluding seeds derived from unobserved pollen transfer, contamination during plant handling, or recording errors. Under an infinite carry-over model, this exclusion resulted in a loss of 11.47% of seeds (85 seeds) and 13.20% of mate pairs. Using a carry-over model with a limit of ten flowers (our biologically realistic hypothesis), seed loss increased to 27.13% (201 seeds), with a 26.73% loss of mate pairs. Note that when we include selfing, the exclusion resulted respectively in a loss of 7.73% of seeds under an infinite carry-over model and of 18.29% of seeds under a carry-over model with a limit of ten flowers. Since bumblebees occasionally exhibit imprecision in their flower visits, we attempted to assign visits to flowers adjacent to those observed, which might account for some unobserved pollen transfer. However, efforts to mitigate data loss by weighting nearby flowers did not effectively resolve these discrepancies. Consequently, we assigned visits strictly to the observed flowers to maintain the dataset's clarity and avoid unnecessary complexity. The mean error on the sum of the components of the variance of reproductive success compared to the actual variance of reproductive success was 21.32% (± 0.16 SE), all analyses combined.

Genetic number of mates

- Male function

Using the genetic mate number as classically used in variance decomposition, we decomposed male reproductive success as classically performed in previous studies (Equation 1; 3–5)

$$RS_m = gMS_m \times FS \times FEC \quad (1)$$

with gMS_m corresponding to the genetic mate number for the male function (encompassing both pre- and post-pollination component) and including individuals with zero mates, FS to the average proportion of seeds sired per mate (post-pollination component, assessed by genotyping seeds to address Fertilization Success), and FEC to the average number of seeds produced by each mate, which represents the partner's fecundity. Pélissié et al. (2012) provided an approximation of the relativized variance in reproductive success (Equation 2; 5).

$$Var(RS_m) \simeq Var(gMS_m) + Var(FS) + Var(FEC) + 2\,Cov(\phi) \quad (2)$$

with each variance component being relativized by their grand mean and $2\,Cov(\phi)$representing the sum of the three pairwise covariances between the main components. Results are available in Figure 1B and S5B for the outcrossed reproductive success. Note that only males (and females in the below analyses) with $gMS > 0$ are considered to compute variance in FS and FEC, variance in these two components are therefore conditional to mate acquisition. Similarly, when focusing on observational mating success, only individuals with $oMS > 0$ are considered to compute variance in the subsequent component.

- Female function

We decomposed female reproductive success into its different components also following classical methods (Equation 3; 3–5).

$$RS_f = gMS_f \times FEC \quad (3)$$

with gMS_f corresponding to the genetic mate number for the female function (encompassing both pre- and post-pollination component) and FEC to the average number of seeds sired per mate, which represents primarily the female function fecundity and secondarily the partner's ability to fertilize ovules and develop seeds. We then obtained the formula for the relativized variance decomposition as follows (Equation 4).

$$Var(RS_f) \simeq Var(gMS_f) + Var(FEC) + 2\,Cov(gMS_f, FEC) \quad (4)$$

Observational number of mates

- Male function

Using the observational mate number, we developed a novel procedure for decomposing male reproductive success as described in Equation 5.

$$RS_m = oMS_m \times VIS \times FS \times FEC \quad (5)$$

with oMS_m corresponding to the observational mate number for the male function (pre-pollination component) and including individuals with zero mate reached, VIS to the average number of flowers visited per mate (pre-pollination component), FS to the average proportion of seeds sired per mate (post-pollination component, assessed by genotyping seeds to address Fertilization Success), and FEC the number of seeds produced per flower visited, which represents the partner's fecundity. In the variance decomposition performed using observational data, we

therefore obtain variance components that correspond strictly to the pre-pollination or to the post-pollination phase, thus enabling the estimation of their relative importance. An approximation of the relativized variance in male reproductive success is then provided in Equation 6:

$$Var\left(RS_m\right) \simeq Var\left(oMS_m\right) + Var\left(VIS\right) + Var\left(FS\right) + Var\left(FEC\right) + 2\,Cov\left(\phi\right) \qquad (6)$$

with $2\,Cov\left(\phi\right)$ representing here the sum of the six pairwise covariances between the main components.

- Female function

Using the observational mate number, we developed a novel procedure for decomposing female reproductive success as described in Equation 7.

$$RS_f = oMS_f \times VIS \times FEC \qquad (7)$$

with oMS_f corresponding to the observational mate number for the female function (pre-pollination component), VIS to the average number of flowers visited per mate (pre-pollination component) and FEC to the average number of seeds sired per mate per flower, which represents primarily the female function fecundity and secondarily the partner’s ability to fertilize ovules and develop seeds.
