# Supplementary material for: Pollinator abundance shapes sexual selection in an angiosperm
Source: PLoS Biol. 2026 Oct 27;24(10):e3003579. doi: 10.1371/journal.pbio.3003579 (PMC13712744; doi:10.1371/journal.pbio.3003579)
Supplement: Supplementary file 4 [file pbio.3003579.s004.pdf]

A

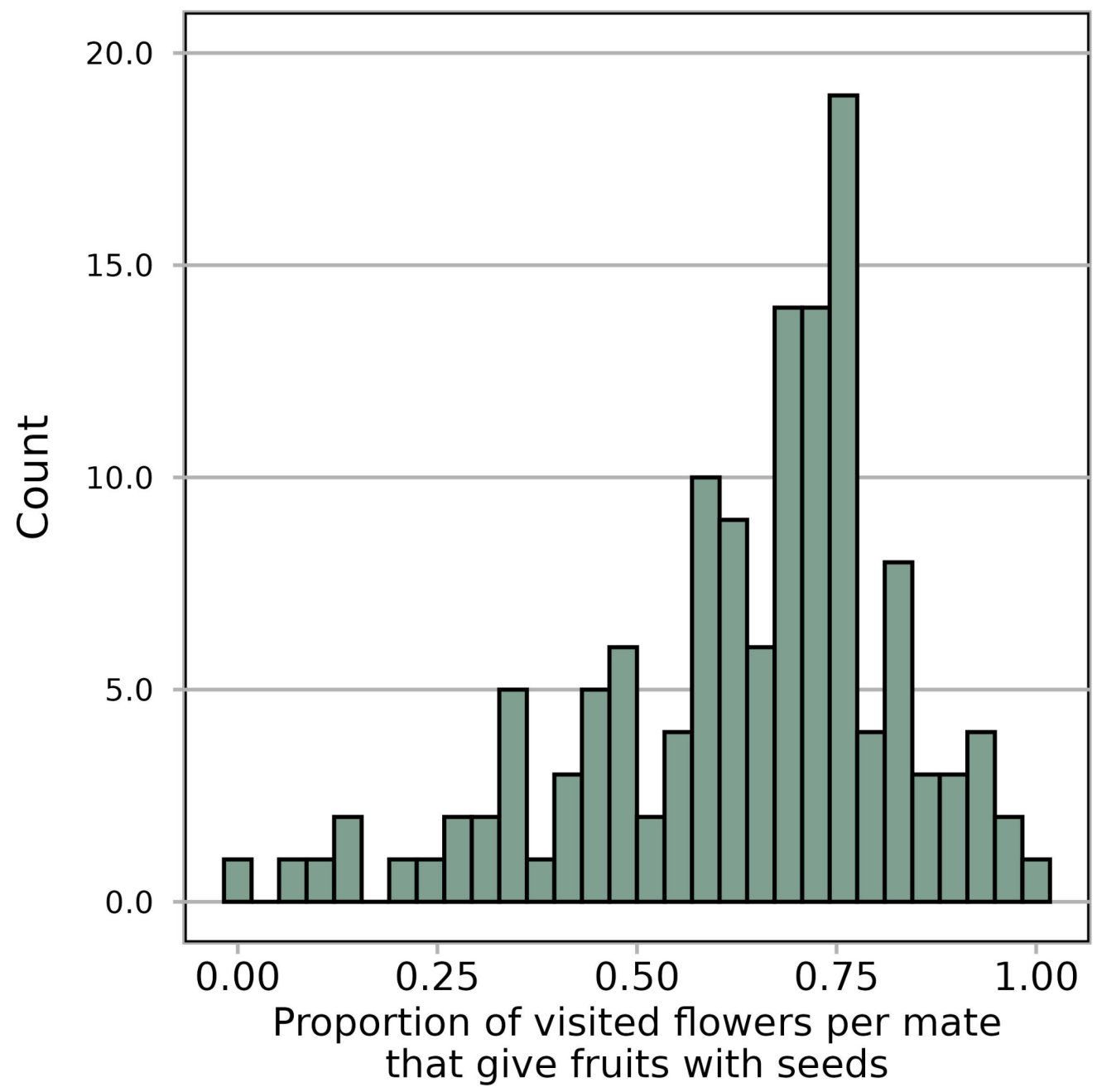

Fig. S1. Distribution of the proportion of flowers visited per mate that give fruits with seeds.
