# Supplementary material for: Pollinator abundance shapes sexual selection in an angiosperm
Source: PLoS Biol. 2026 Oct 27;24(10):e3003579. doi: 10.1371/journal.pbio.3003579 (PMC13712744; doi:10.1371/journal.pbio.3003579)
Supplement: Supplementary file 5 [file pbio.3003579.s005.pdf]

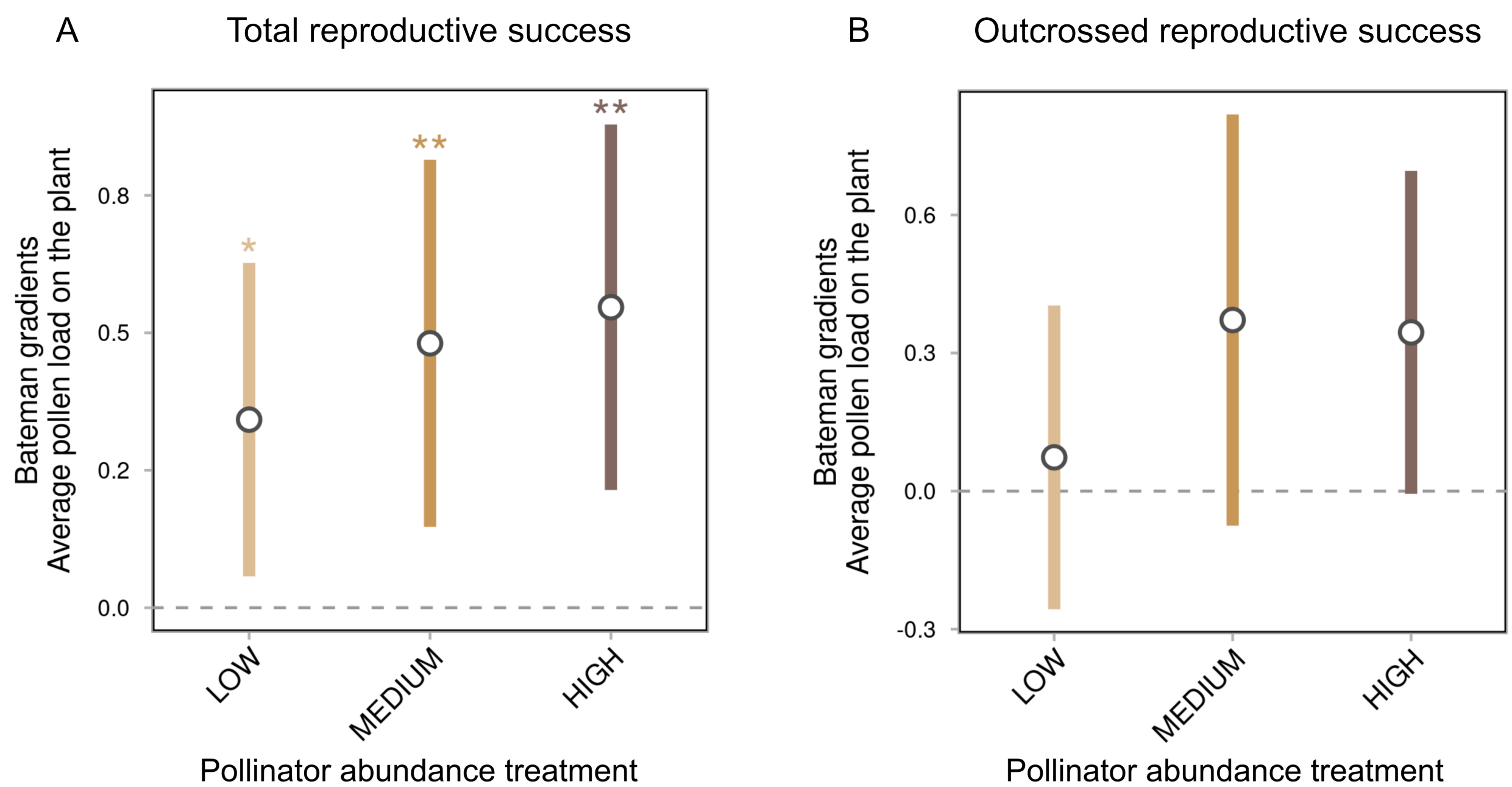

Fig. S2. Effects of the pollinator abundance treatment on the Bateman gradients on pollen loads for both reproductive successes. Bateman gradients (estimate ± 95% confidence intervals) on average pollen load on the plant are displayed for each pollinator abundance treatment (low, medium, or high abundance), using total (A) or outcrossed (B) female reproductive success. Bateman gradients differing significantly from zero are indicated above each estimate in the corresponding color, with stars corresponding to the p-value from t-tests (˙ < .10, * < .05, ** < .01, *** < .001). Significant treatment, sex or interaction effects, as identified by likelihood ratio tests (Table S2), are indicated in this figure by black lines with their associated p-values from t-tests for pairwise comparison between modalities.
