# Supplementary material for: Pollinator abundance shapes sexual selection in an angiosperm
Source: PLoS Biol. 2026 Oct 27;24(10):e3003579. doi: 10.1371/journal.pbio.3003579 (PMC13712744; doi:10.1371/journal.pbio.3003579)
Supplement: Supplementary file 6 [file pbio.3003579.s006.pdf]

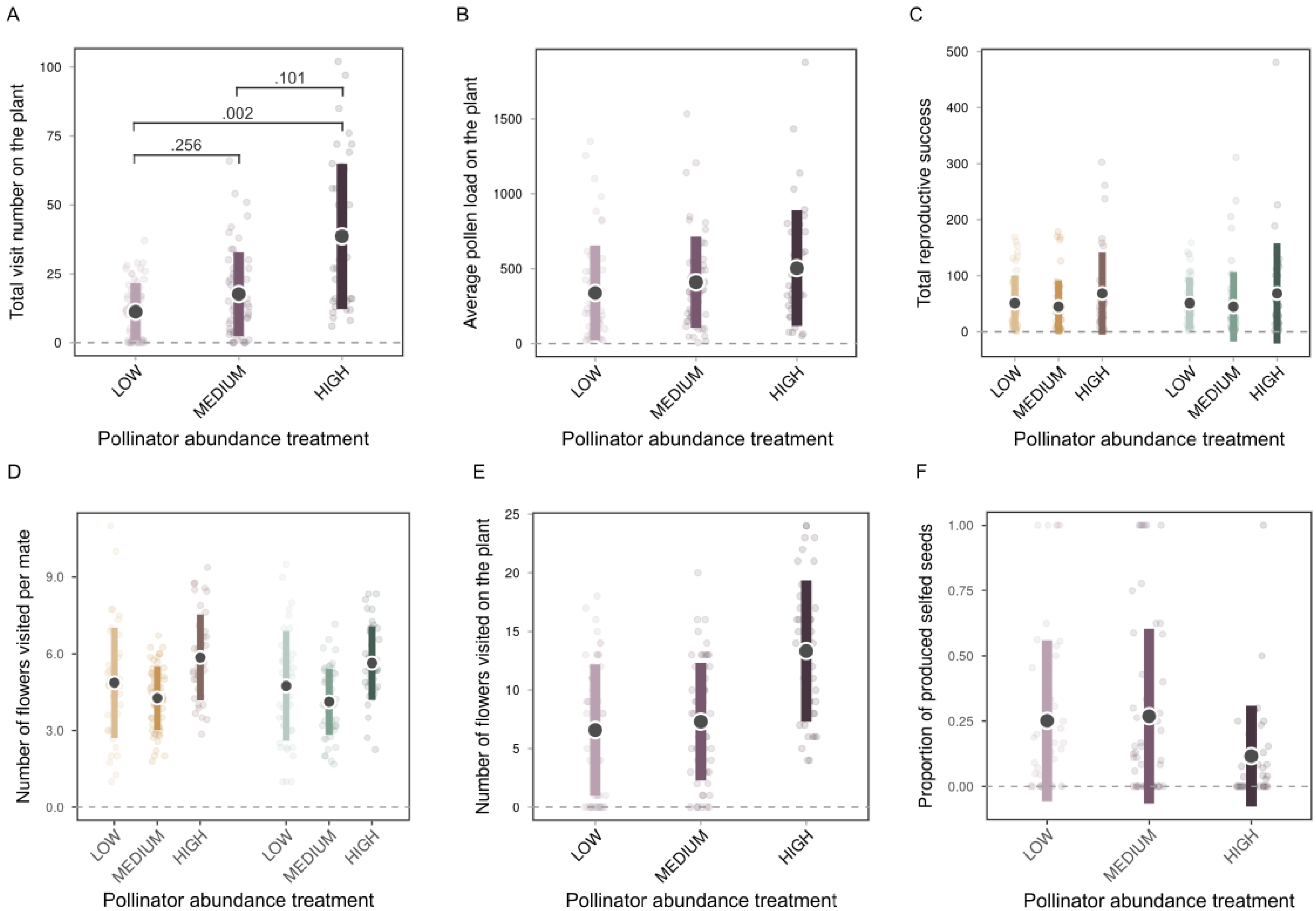

A
Total visit number on the plant
100
75
50
25
0
.101
.002
.256
LOW
MEDIUM
HIGH
Pollinator abundance treatment
B
Average pollen load on the plant
1500
1000
500
0
LOW
MEDIUM
HIGH
Pollinator abundance treatment
C
Total reproductive success
500
400
300
200
100
0
LOW
MEDIUM
HIGH
LOW
MEDIUM
HIGH
Pollinator abundance treatment
D
Number of flowers visited per mate
9.0
6.0
3.0
0.0
LOW
MEDIUM
HIGH
LOW
MEDIUM
HIGH
Pollinator abundance treatment
E
Number of flowers visited on the plant
25
20
15
10
5
0
LOW
MEDIUM
HIGH
Pollinator abundance treatment
F
Proportion of produced selfed seeds
1.00
0.75
0.50
0.25
0.00
LOW
MEDIUM
HIGH
Pollinator abundance treatment

Fig. S3. Effect of pollinator abundance on pollinator visitation, pollen loads, and plant reproductive success. The impact of pollinator abundance treatment (low, medium, or high) was assessed on the following variables (mean ± standard deviation): (A) total number of visits to the plant, (B) average pollen load on the plant, (C) total reproductive success, (D) number of flowers visited per mate, (E) number of flowers visited on the plant and (F) proportion of produced selfed seeds. When applicable, variables associated with the female function are shown in shades of orange, those related to the male function in shades of green, and variables shared by both sexual functions in shades of purple. Significant treatment effects, identified by likelihood ratio tests (Table S5), are indicated by p-values from post-hoc Tukey tests for pairwise comparisons between treatment levels shown by black connecting lines.
