# Supplementary material for: Pollinator abundance shapes sexual selection in an angiosperm
Source: PLoS Biol. 2026 Oct 27;24(10):e3003579. doi: 10.1371/journal.pbio.3003579 (PMC13712744; doi:10.1371/journal.pbio.3003579)
Supplement: Supplementary file 7 [file pbio.3003579.s007.pdf]

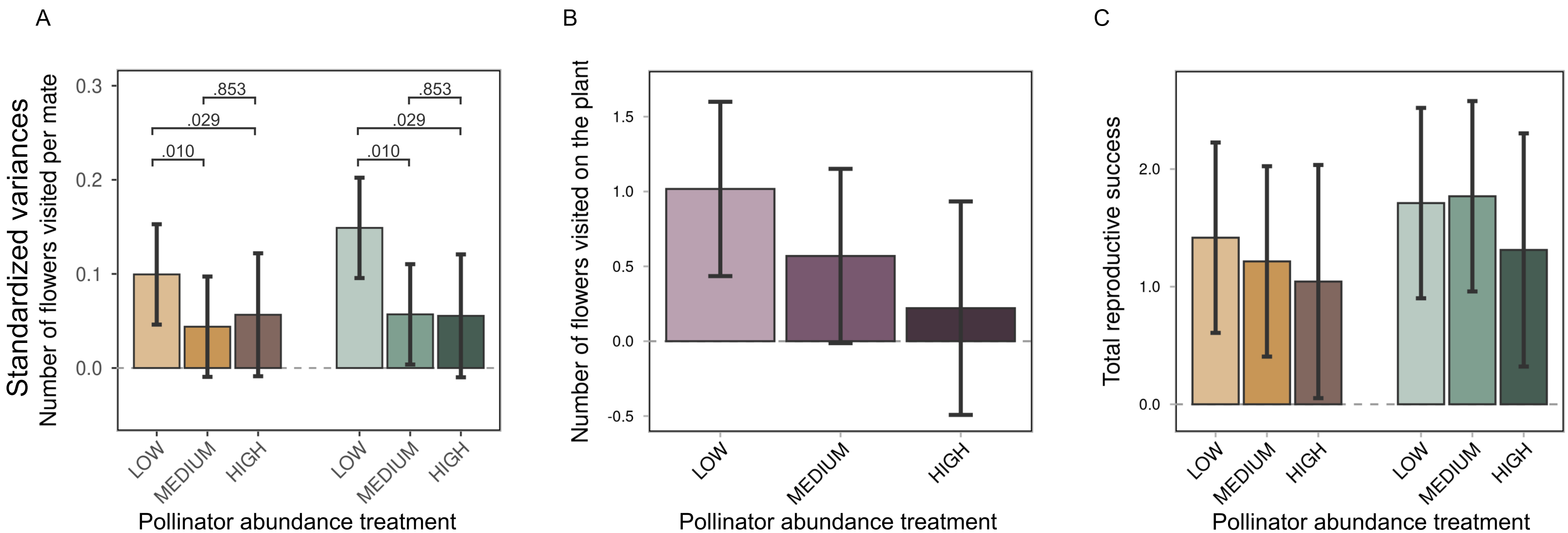

Fig. S4. Effects of pollinator abundance on the opportunities for selection. The impact of pollinator abundance treatment (low, medium, or high) was evaluated for the following measures of opportunity for selection (mean ± 95% confidence intervals): (A) number of flowers visited per mate, (B) number of flowers visited on the plant, and (C) total reproductive success. When applicable, variables related to the female function are shown in shades of orange, those related to the male function in shades of green, and variables shared by both sexual functions in shades of purple. Significant treatment effects, as identified by likelihood ratio tests (Table S6), are indicated by p-values from t-tests conducted for pairwise comparisons between treatment levels shown by black connecting lines.
