# Supplementary material for: Pollinator abundance shapes sexual selection in an angiosperm
Source: PLoS Biol. 2026 Oct 27;24(10):e3003579. doi: 10.1371/journal.pbio.3003579 (PMC13712744; doi:10.1371/journal.pbio.3003579)
Supplement: Supplementary file 8 [file pbio.3003579.s008.pdf]

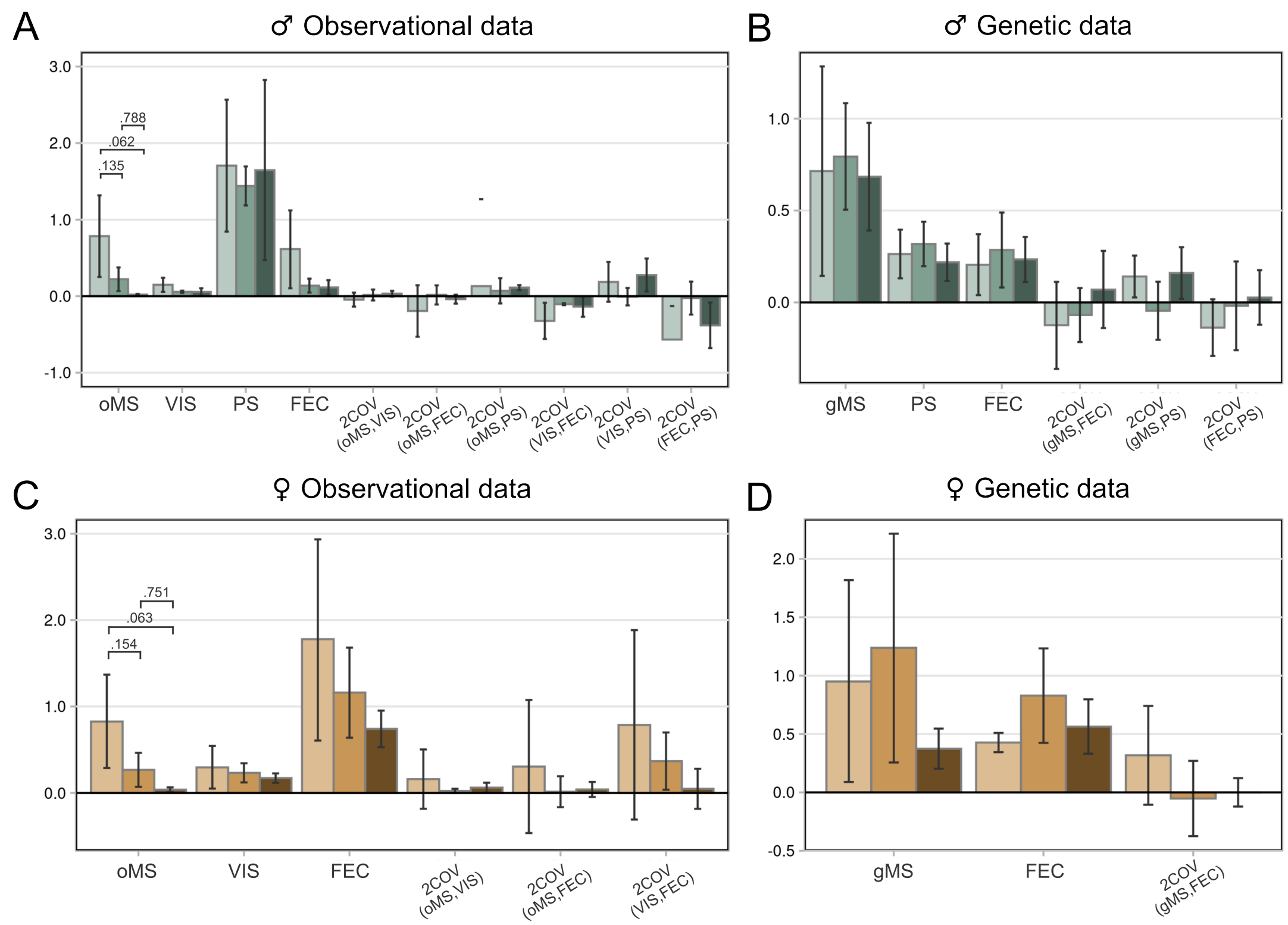

Fig. S5. Effect of pollinator abundance treatment (low: light color, medium: medium color, high: dark color) on variance components of outcrossed reproductive success (mean ± 95% bootstrapped confidence intervals), including covariances, in males (A, B) and females (C, D). The analysis focuses on the genetic number of mates (A, C) and the observational number of mates (B, D). When variance components differed significantly between pollinator treatments, as indicated by likelihood ratio tests on nested models (Table S7), adjusted p-values from pairwise comparisons using post-hoc Tukey's HSD tests are displayed by black connecting lines.

Abbreviations: (A) oMS: observational number of mates, VIS: number of flowers visited per mate, FS: proportion of seeds sired per mate, FEC: number of seeds produced per flower visited, (B) gMS: genetic number of mates, FS: proportion of seeds sired per mate, FEC: number of seeds produced per mate, (C) oMS: observational number of mates, FEC: number of seeds sired per mate per flower visited, VIS: number of flowers visited per mate, (D) gMS: genetic number of mates, FEC: number of seeds sired per mate.
