# Supplementary material for: Pollinator abundance shapes sexual selection in an angiosperm
Source: PLoS Biol. 2026 Oct 27;24(10):e3003579. doi: 10.1371/journal.pbio.3003579 (PMC13712744; doi:10.1371/journal.pbio.3003579)
Supplement: Supplementary file 9 [file pbio.3003579.s009.pdf]

Table S1. Results of likelihood ratio tests testing for the main component of reproductive successes for both sexual functions according to the data type employed for variance decomposition.

	Data type employed for variance decomposition	Reproductive success calculation	*N*	Likelihood ratio tests - LRT									Comparison	Post-hoc Tukey's HSD		LRT per treatment			
				Interaction effect			Component identity effect			Treatment effect				Pairwise differences	Adjusted *p*-value	Component identity effect			
				χ^2	*df*	*p*-value	χ^2	*df*	*p*-value	χ^2	*df*	*p*-value				*N*	χ^2	*df*	*p*-value
Females	Observational	Outcrossed	48	4.25	4	.373	23.09	2	**<.001**	4.22	2	.121	FEC - oMS	0.87 ± 0.20	**<.001**				
													FEC - VIS	1.00 ± 0.20	**<.001**				
													oMS - VIS	0.18 ± 0.20	.645				
	Genetic	Total	32	7.09	2	**.029**							LOW: FEC - gMS	<.001 ± 0.30		12	<.001	1	.975
													MEDIUM: FEC - gMS	1.00 ± 0.36		12	6.51	1	**.011**
													HIGH: FEC - gMS	0.41 ± 0.16		8	7.34	1	**.007**
		Outcrossed	30	1.19	2	.551	1.11	1	.292	2.82	2	.244	FEC - gMS	-0.23 ± 0.29	.340				
Males	Observational	Outcrossed	64	4.01	6	.675	51.10	3	**<.001**	3.29	2	.192	FEC - oMS	-0.072 ± 0.18	.979				
													FEC - FS	-1.28 ± 0.18	**<.001**				
													FEC - VIS	0.22 ± 0.18	.622				
													oMS - FS	-1.21 ± 0.18	**<.001**				
													oMS - VIS	0.29 ± 0.18	.384				
													FS - VIS	1.50 ± 0.18	**<.001**				
	Genetic	Total	48	2.81	4	.590	5.72	2	*.057*	0.55	2	.761	FEC - gMS	-0.24 ± 0.13	.139				
													FEC - FS	0.03 ± 0.13	.975				
													gMS - FS	0.27 ± 0.13	.091				
		Outcrossed	45	0.09	4	.999	19.69	2	**<.001**	0.72	2	.697	FEC - gMS	-0.49 ± 0.12	**<.001**				
													FEC - FS	-0.03 ± 0.12	.968				
													gMS - FS	0.46 ± 0.12	**.001**				

Results of likelihood ratio tests testing for the effect of the variance component identity, the pollinator abundance treatment and their interaction on the amount of variance in female and male reproductive success, for different data type (genetic or observational) and reproductive success calculation (outcrossed or total), as well as pairwise differences (± standard-errors) between each variance component of reproductive success and their significance determined by adjusted p-values from post-hoc Tukey's HSD tests. Pairwise differences in comparison between variance components are shown with the first term as a reference. Our analysis of variance decomposition of male reproductive success shows that the genetic number of mates (gMS) was a significantly larger component compared to both the fertilization success (FS) and the fecundity of the partner (FEC), both when computed on outcrossed or total reproductive success. On the contrary, when using observational data, the proportion of seeds sired per mate (FS) was a significantly larger component compared to the number of seeds produced per flower visited (FEC), the observational number of mates (oMS) and the number of flowers visited per mate (VIS). For variance decomposition of female reproductive success, the genetic number of mates (gMS) was of similar contribution to the number of seeds sired per mate (FEC), except when computed on total reproductive success for the medium and high pollinator abundance treatments. In those cases, the number of seeds sired per mate (FEC) contributed more to reproductive success than the genetic number of mates (gMS). When using observational data, the number of seeds sired per mate per flower visited (FEC) was a significantly larger component compared to the observational number of mates (oMS) and the number of flowers visited per mate (VIS).
