# Supplementary material for: Pollinator abundance shapes sexual selection in an angiosperm
Source: PLoS Biol. 2026 Oct 27;24(10):e3003579. doi: 10.1371/journal.pbio.3003579 (PMC13712744; doi:10.1371/journal.pbio.3003579)
Supplement: Supplementary file 10 [file pbio.3003579.s010.pdf]

Table S2. Effects of pollinator abundance treatment, sex (when appropriate) and their interaction on estimated Bateman gradients.

	Variable	*N*	Likelihood ratio tests								
			Interaction effet			Sex effect			Treatment effect		
			χ^2	*df*	*p*-value	χ^2	*df*	*p*-value	χ^2	*df*	*p*-value
Bateman gradients based on total reproductive success	Observational number of mates	250	0.46	2	.793	8.44	1	**.004**$^{x \in [7.\infty[}$	5.35	2	*.069*
	Genetic number of mates	268	2.64	2	.267	6.58	1	**.010**	3.63	2	.163
	Number of flowers visited per mate	250	2.47	2	.290	0.09	1	.925	5.26	2	*.072*
	Number of flowers visited on the plant	272	0.16	2	.922	0.02	1	.883	5.99	2	**.050**
	Total visit number	272	0.35	2	.841	<.01	1	.970	6.69	2	**.035**
	Average pollen load *	137							0.94	2	.624
Bateman gradients based on outcrossed reproductive success	Observational number of mates	220	0.45	2	.800	5.17	1	.134	7.84	2	**.020**$^{x \in [8.17]}$
	Genetic number of mates	228	1.19	2	.552	2.08	1	.149	6.67	2	**.036**
	Number of flowers visited per mate	220	5.17	2	*.075*	0.02	1	.880	5.82	2	*.055*
	Number of flowers visited on the plant	228	0.45	2	.800	0.84	1	.359	6.91	2	**.032**
	Total visit number	220	0.47	2	.800	0.11	1	.741	5.99	2	**.050**
	Average pollen load *	113							1.74	2	.420

Results of likelihood ratio tests are shown with sample size (*N*), chi-squared statistic (χ^2), degrees of freedom (*df*) and associated *p*-value. For variables relying on the computation of pollen carry-overs (defined as the successive number of flowers visited for which pollen export/import remains effective), the range of carry-over values x for which the result remains significant is specified in the form $x \in [a,b]$, where a and b denote, respectively, the shortest and longest pollen dispersal kernels for which results remained significant.

* Results for Bateman gradients based on pollen loads are presented only for female reproductive success, as the amount of pollen deposited on stigmas can influence seed production solely via the female function.
