# Supplementary material for: Pollinator abundance shapes sexual selection in an angiosperm
Source: PLoS Biol. 2026 Oct 27;24(10):e3003579. doi: 10.1371/journal.pbio.3003579 (PMC13712744; doi:10.1371/journal.pbio.3003579)
Supplement: Supplementary file 11 [file pbio.3003579.s011.pdf]

Table S3. Relationships between male and female fitness components assessed for the three tested pollinator abundances separately.

	LOW					MEDIUM					HIGH				
	Estimate	*N*	χ^2	*df*	*p*-value	Estimate	*N*	χ^2	*df*	*p*-value	Estimate	*N*	χ^2	*df*	*p*-value
Observational number of mates	0.04 ± 0.16	44	0.07	1	.800	0.51 ± 0.14	54	12.21	1	**<.001**$^{x \in [1.12]}$	0.52 ± 0.18	40	7.71	1	**0.005**$^{x \in [1.12]}$
Genetic number of mates	0.10 ± 0.13	42	0.62	1	.432	0.17 ± 0.16	50	1.17	1	.280	0.05 ± 0.11	33	0.20	1	.658
Outcrossed reproductive success	-0.07 ± 0.26	31	0.08	1	.773	-0.20 ± 0.16	37	1.59	1	.207	-0.23 ± 0.18	29	1.74	1	.186

Regressions (estimates ± standard-errors) for outcrossed reproductive success, observational and genetic number of mates between male and female sexual function computed for each pollinator abundance. Linear mixed-effects models were used for the regression of male-function values on female-function values, with a random effect of the experimental population. Results of likelihood ratio tests on the female function values are shown with sample size (*N*), chi-squared statistic (χ^2), degrees of freedom (*df*) and associated *p*-value. For variables relying on the computation of pollen carry-overs (defined as the successive number of flowers visited for which pollen export/import remains effective), the range of carry-over values *x* for which the result remains significant is specified in the form $x \in [a,b]$, where a and b denote, respectively, the shortest and longest pollen dispersal kernels for which results remained significant.
