# Supplementary material for: Pollinator abundance shapes sexual selection in an angiosperm
Source: PLoS Biol. 2026 Oct 27;24(10):e3003579. doi: 10.1371/journal.pbio.3003579 (PMC13712744; doi:10.1371/journal.pbio.3003579)
Supplement: Supplementary file 12 [file pbio.3003579.s012.pdf]

Table S4. Results of likelihood ratio tests testing for the cross-sex effects for the Bateman gradients using either the genetic number of mates or the observational number of mates, for each sexual function.

		LOW				MEDIUM				HIGH			
		N	χ^2	*df*	*p*-value	*N*	χ^2	*df*	*p*-value	*N*	χ^2	*df*	*p*-value
Observational number of mates	Female	35	0.32	1	.574	42	0.37	1	.542	34	0.01	1	.913
	Male	34	0.46	1	.498	43	0.95	1	.330	32	0.45	1	.500
Genetic number of mates	Female	33	0.52	1	.472	40	0.14	1	.708	32	0.06	1	.803
	Male	35	2.74	1	.098	41	1.70	1	.192	29	1.26	1	.261

Cross-sex effects were explored for each sex and treatment separately, the regression of outcrossed reproductive success of the focal sex on both (i) the number of mates (either observational or genetic) of the focal sex and (ii) the number of mates (either observational or genetic) of the opposite sex. The latter term represents what is referred to as the cross-sex effect [6]. Results of likelihood ratio tests on the cross-sex effect are shown with sample size (*N*), chi-squared statistic (χ^2), degrees of freedom (*df*) and associated *p*-value.
