# Supplementary material for: Pollinator abundance shapes sexual selection in an angiosperm
Source: PLoS Biol. 2026 Oct 27;24(10):e3003579. doi: 10.1371/journal.pbio.3003579 (PMC13712744; doi:10.1371/journal.pbio.3003579)
Supplement: Supplementary file 13 [file pbio.3003579.s013.pdf]

Table S5. Results of likelihood ratio tests testing for the effects of pollinator abundance treatment, sex, and their interaction, on the measured variables.

			Likelihood ratio tests								
Variables	Distribution	*N*	Treatment effect			Sex effect			Interaction effect		
			χ^2	df	*p*-value	χ^2	df	*p*-value	χ^2	df	*p*-value
Total reproductive success [(1)]	Poisson	270	2.49	2	.288	0.17	1	.681	0.46	2	.793
Outcrossed reproductive success [(1)]	Poisson	228	3.14	2	.208	0.02	1	.902	0.19	2	.911
Observational number of mates	Poisson	276	8.66	2	**.013** $^{x \in [1.50]}$	0.00	1	1.000 [(2)]	0.00	2	1.000 [(2)]
Genetic number of mates	Poisson	268	3.35	2	.187	0.05	1	.816	0.04	2	.981
Number of flowers visited per mate	Gaussian	272	4.17	2	.124	1.75	1	.186	0.11	2	.948
Number of flowers visited on the plant	Poisson	160	5.78	2	.056						
Total number of visits on the plant	Poisson	160	8.97	2	**.011**						
Average pollen load on the plant	Poisson	379	1.59	2	.451						
Proportion of produced selfed seeds	Binomial	143	0.10	2	.105						

Results of likelihood ratio tests are shown with sample size (*N*), chi-squared statistic (χ^2), degrees of freedom (*df*) and associated *p*-value. For variables that involved the computation of pollen carry-over (defined as the number of successive flowers visited during which pollen export/import remains effective), the set of carry-over values *x* for which the result remains significant is specified in the form $x \in [a,b]$, where a and b denote, respectively, the shortest and longest pollen dispersal kernels for which results remained significant.

(1) Including non-reproducing individuals did not alter the results. There was no effect of sex, treatment, or their interaction on either total or outcrossed reproductive success.

(2) The observational number of mates is, on average, identical between the male and female functions, resulting in an absolute null effect of sex and its interaction with treatment. This was expected because the directionality of pollinator visits moving from one plant to the next necessarily leads to symmetry in observed numbers of sexual partners obtained by the male or female function, with the notable exception of the first visit in the sequence (i.e. effective for the male function only) and the last visit (i.e. effective for the female function only). In our analysis, the effects of the first and last visits are minimal because the first and last plants visited by a pollinator during observation sessions were always revisited at least once during the sequence. This finding holds true for all carry-over values explored, ranging from 1 to infinity.
