# Supplementary material for: Pollinator abundance shapes sexual selection in an angiosperm
Source: PLoS Biol. 2026 Oct 27;24(10):e3003579. doi: 10.1371/journal.pbio.3003579 (PMC13712744; doi:10.1371/journal.pbio.3003579)
Supplement: Supplementary file 14 [file pbio.3003579.s014.pdf]

Table S6. Results of likelihood ratio tests on the effects of sex, treatment, and their interaction on the opportunities for selection (*I*) and the opportunities for sexual selection (I_s).

	Index	*N*	Likelihood ratio tests								
			Treatment effect			Sex effect			Interaction effect		
			χ^2	*df*	*p*-value	χ^2	*df*	*p*-value	χ^2	*df*	*p*-value
Opportunity for selection	*I* – Total reproductive success	32	0.60	2	.741	2.60	1	.107	0.33	2	.848
	I – Outcrossed reproductive success	32	1.35	2	.510	2.27	1	.132	3.26	2	.196
Opportunity for sexual selection	I_s – Observational number of mates	32	8.32	2	**.016**$^{x \in [1.12]}$	0.03	1	.854	0.69	2	.710
	I_s – Genetic number of mates	32	3.42	2	.397	0.92	1	.336	1.85	2	.181
	I_s – Number of flowers visited per mate	32	8.87	2	**.012**$^{x \in [5.\infty[}$	1.13	1	.287	0.97	2	.616
	I_s – Number of flowers visited on the plant	16	1.58 (*SS*)	2	.224						

Results of likelihood ratio tests are shown with sample size (*N*), chi-squared statistic (χ^2, or the sum of squares statistic *SS* when relevant), degrees of freedom (*df*) and associated *p*-value. To estimate the opportunity for selection and sexual selection, all individuals were included in the dataset. Individuals that did not reproduce or reproduced only through selfing were assigned null values for both reproductive and mating success. For the variables relying on the computation of pollen carry-overs (defined as the successive number of flowers visited during which pollen export/import remains effective), the range of carry-over values *x* for which the result remains significant is specified in the form $x \in$ [a,b], where a and b denote, respectively, the shortest and longest pollen dispersal kernels for which results remained significant.
