# Supplementary material for: Pollinator abundance shapes sexual selection in an angiosperm
Source: PLoS Biol. 2026 Oct 27;24(10):e3003579. doi: 10.1371/journal.pbio.3003579 (PMC13712744; doi:10.1371/journal.pbio.3003579)
Supplement: Supplementary file 15 [file pbio.3003579.s015.pdf]

Table S7. Results of likelihood ratio tests testing for the effect of pollinator abundance treatment on the variance components of the female and male reproductive successes.

	Data type	Reproductive success calculation	Variance component	*N*	*SS*	*df*	*p*-value
Females	Observational	Outcrossed	oMS – observational number of mates	16	1.72	2	**.027**
			VIS – number of flowers visited per mate	16	0.04	2	.704
			FEC – number of seeds sired per mate per flower visited	16	2.72	2	.318
			2COV(oMS, VIS)	16	0.06	2	.712
			2COV(oMS, FEC)	16	0.30	2	.721
			2COV(VIS, FEC)	16	1.37	2	.489
	Genetic	Total	gMS – genetic number of mates	16	0.41	2	.449
			FEC – number of seeds sired per mate	16	2.76	2	**.011**
			2COV (gMS, FEC)	16	0.71	2	.223
		Outcrossed	gMS – genetic number of mates	15	2.52	2	.462
			FEC – number of seeds sired per mate	15	1.80	2	.217
			2COV (gMS, FEC)	15	0.46	2	.324
Males	Observational	Outcrossed	oMS – observational number of mates	16	1.65	2	**.024**
			VIS – number of flowers visited per mate	16	0.03	2	.103
			FS – proportion of seeds sired per mate	16	0.23	2	.895
			FEC – number of seeds produced per flower visited	16	0.88	2	.105
			2COV(oMS, VIS)	16	0.02	2	.420
			2COV(oMS, FEC)	16	0.14	2	.476
			2COV(oMS, FS)	16	0.01	2	.994
			2COV(VIS, FEC)	16	0.16	2	.175
			2COV(VIS, FS)	16	0.22	2	.229
			2COV(FEC, FS)	16	0.90	2	.104
	Genetic	Total	gMS – genetic number of mates	16	0.20	2	.675
			FS – proportion of seeds sired per mate	16	0.08	2	.501
			FEC – number of seeds produced per mate	16	0.10	2	.605
			2 COV (gMS, FEC)	16	0.09	2	.868
			2 COV (gMS, FS)	16	0.06	2	.624
			2 COV (FEC, FS)	16	0.01	2	.931

When selfing is included in reproductive success, the focal individual itself must be counted as a sexual partner to obtain the correct total sum of variance components. Results are shown with sample size (*N*), followed by the sum of squares statistic (*SS*), the degrees of freedom (*df*) and the associated p-value. For variance decomposition using genetic data, we reported statistics for both total and outcrossed reproductive success only for the female function, as the results were similar for both reproductive success calculation in the male function.
