# Supplementary material for: Pollinator abundance shapes sexual selection in an angiosperm
Source: PLoS Biol. 2026 Oct 27;24(10):e3003579. doi: 10.1371/journal.pbio.3003579 (PMC13712744; doi:10.1371/journal.pbio.3003579)
Supplement: Supplementary file 16 [file pbio.3003579.s016.pdf]

Table S8. Results of likelihood ratio tests for model comparison on the relationship between genetic and observational number of mates using reproductive skew measures (Q_{gen} vs. Q_{obs}).

Response	Fixed effect	Heteroscedasticity	Test	χ^2	*p*-value	Conclusion
(1) Q_{gen}	Q_{obs} * Treatment	Yes				
(2) Q_{gen}	Q_{obs} * Treatment	No	(2) vs (1)	16.65	<.001	High heteroscedasticity
(3) Q_{gen}	Q_{obs} + Treatment	Yes	(3) vs (1)	0.24	.885	Similar slopes between treatment modalities
(4) Q_{gen}	Q_{obs}	Yes	(4) vs (3)	1.39	.845	Similar intercepts between treatment modalities
(5) Q_{gen}	Intercept only	Yes	(5) vs (4)	11.39	<.001	Q_{obs} slope differ from 1
(6) Q_{gen}	Regression line forced through point $Q_{obs} = Q_{gen}$ for largest observed Q_{obs}.	Yes	(6) vs (4)	10.56	<.01	$Q_{gen} > Q_{obs}$ for high Q_{obs} and conversely for low Q_{obs}.

The response variable and the fixed effects included in the linear mixed-effect models are indicated. Heteroscedasticity of residuals was accounted for (Yes) or not (No) by fitting the residual variance as a function of the number of genotyped seeds from which each Q_{gen} value was obtained. Likelihood ratio test results comparing models are reported, including the chi-squared statistic (χ^2), associated p-value, and a verbal interpretation of each result.
