# Supplementary material for: Pollinator abundance shapes sexual selection in an angiosperm
Source: PLoS Biol. 2026 Oct 27;24(10):e3003579. doi: 10.1371/journal.pbio.3003579 (PMC13712744; doi:10.1371/journal.pbio.3003579)
Supplement: Supplementary file 17 [file pbio.3003579.s017.pdf]

Table S9. Properties of the eight nuclear microsatellites used for individual allelic characterization and paternity analysis.

Marker	Range size (bp)	Fluorochrome	Repeat motif	Allele number	Forward sequence	Reverse sequence
72	190-225	PET	$(TCT)_7$	4	GGCGGATATCTAGAGAGAGGAGA	CGGAGCTATACGTCTGTGAGAAG
87	130-170	VIC	$(CT)_{11}$	5	CCGAGGAAGGGAAGTTGTAA	AGGAGTCCAACCAGAGAACG
198	210-270	VIC	$(AG)_8$	8	CCTCCGTGAAGAGGTACTCG	GTCGCCTCTCCTTCTCTCTCT
374	260-330	NED	$(TTC)_{11}$	6	TTCCTTATTGGGATGTCGTG	CTCGTTGTTGAGGAAGCAAA
396	160-180	NED	$(AC)_6$	2	CGCTTAGCACTGTCTCCTTG	CTCTGTCGTGGTGATTCTGC
489	270-320	PET	$(AAC)_7$	3	AACCTTCTCAGGCAAACGAT	AGTTCATCGAAAGGGTTGCT
650	150-185	PET	$(TC)_8$	3	TCCACCATCTCATTCTTCCA	TGCCCTCTTTCCAATAATCC
790	210-250	NED	$(AT)_{20}$	7	AGAAGCAGCGAATATCGTG	GCTCCACAACACAAACAATTC
