# Supplementary material for: Pollinator abundance shapes sexual selection in an angiosperm
Source: PLoS Biol. 2026 Oct 27;24(10):e3003579. doi: 10.1371/journal.pbio.3003579 (PMC13712744; doi:10.1371/journal.pbio.3003579)
Supplement: Supplementary file 18 [file pbio.3003579.s018.pdf]

Table S10. Results of likelihood ratio tests testing for the effects of pollinator abundance treatment, sex, and their interaction, on Jones indexes.

		Index	N	Likelihood ratio tests								
				Treatment effect			Sex effect			Interaction effect		
				χ^2	*df*	*p*-value	χ^2	*df*	*p*-value	χ^2	*df*	*p*-value
Jones Index	Bateman gradients based on total reproductive success	s'_{max} – Observational number of mates	32	0.33	2	.848	0.12	1	.732	1.48	2	.477
		s'_{max} – Genetic number of mates	32	1.36	2	.506	4.86	1	**.028**	3.74	2	.154
		s'_{max} – Number of flowers visited per mate	32	3.32	2	.190	1.10	1	.295	3.34	2	.189
		s'_{max} – Number of flowers visited on the plant	32	0.49	2	.782	0.05	1	.819	0.08	2	.962
	Bateman gradients based on outcrossed reproductive success	s'_{max} – Observational number of mates	30	1.34	2	.512	0.29	1	.587	1.78	2	.411
		s'_{max} – Genetic number of mates	30	0.67	2	.714	**<.01**	1	.994	4.09	2	.130
		s'_{max} – Number of flowers visited per mate	30	2.86	2	.239	0.09	1	.767	6.79	2	**.034**$^{x \in [7.11]}$
		s'_{max} – Number of flowers visited on the plant	30	2.11	2	.348	0.24	1	.626	0.80	2	.672

Results of likelihood ratio tests on the effects of sex, treatment, and their interaction on the Jones Index (s'_{max}). Jones Indexes were estimated using observational and genetic mate numbers, as well as variables reflecting the intensity of pollinator contacts per mate and the focal plant's attractiveness, based on Bateman gradients using total or outcrossed reproductive success. Results of likelihood ratio tests are shown with sample size (N), chi-squared statistic (χ^2), degrees of freedom (*df*) and associated *p*-value. One population was excluded from the dataset when estimating the Jones Index (based on outcrossed reproductive success) because only two individuals in this population reproduced via outcrossing. Specifically, the Bateman gradient (a component of the Jones Index) requires non-null reproductive success to avoid an artificial positive correlation. This is because at least one sexual partner is necessary to produce outcrossed seeds. As a result, the Bateman gradient - and consequently the Jones Index - could not be estimated for this population, For the variables relying on the computation of pollen carry-overs (defined as the successive number of flowers visited during which pollen export/import remains effective), the range of carry-over values x for which the result remains significant is specified in the form $x \in$ [a,b], where a and b denote, respectively, the shortest and longest pollen dispersal kernels for which results remained significant.
